# miRNAs in multiple myeloma – a survival relevant complex regulator of gene expression

**DOI:** 10.18632/oncotarget.5381

**Published:** 2015-10-12

**Authors:** Anja Seckinger, Tobias MeiΔner, Jérôme Moreaux, Vladimir Benes, Jens Hillengass, Mirco Castoldi, Jürgen Zimmermann, Anthony D. Ho, Anna Jauch, Hartmut Goldschmidt, Bernard Klein, Dirk Hose

**Affiliations:** ^1^ Medizinische Klinik V, Universitätsklinikum Heidelberg, Heidelberg, Germany; ^2^ Department of Molecular and Experimental Medicine, Avera Cancer Institute, La Jolla, CA, USA; ^3^ Centre Hospitalier Universitaire Montpellier, Hôpital Saint-Eloi, Montpellier, France; ^4^ European Molecular Biology Laboratory, Heidelberg, Germany; ^5^ Klinik für Gastroenterologie, Hepatologie und Infektiologie, Universitätsklinikum Düsseldorf, Düsseldorf, Germany; ^6^ Institut für Humangenetik, Universität Heidelberg, Heidelberg, Germany; ^7^ Nationales Centrum für Tumorerkrankungen, Heidelberg, Germany

**Keywords:** miRNA, multiple myeloma, gene expression profiling, survival

## Abstract

**Purpose:**

microRNAs regulate gene-expression in biological and pathophysiological processes, including multiple myeloma. Here we address i) What are the number and magnitude of changes in miRNA-expression between normal plasma cells and myeloma- or MGUS-samples, and the latter two? ii) What is the biological relevance and how does miRNA-expression impact on gene-expression? iii) Is there a prognostic significance, and what is its background?

**Experimental design:**

Ninety-two purified myeloma-, MGUS-, normal plasma cell- and myeloma cell line-samples were investigated using miChip-arrays interrogating 559 human miRNAs. Impact on gene-expression was assessed by Affymetrix DNA-microarrays in two cohorts of myeloma patients (*n* = 677); chromosomal aberrations were assessed by iFISH, survival for 592 patients undergoing up-front high-dose chemotherapy.

**Results:**

Compared to normal plasma cells, 67/559 miRNAs (12%) with fold changes of 4.6 to −3.1 are differentially expressed in myeloma-, 20 (3.6%) in MGUS-samples, and three (0.5%) between MGUS and myeloma. Expression of miRNAs is associated with proliferation, chromosomal aberrations, tumor mass, and gene expression-based risk-scores. This holds true for target-gene signatures of regulated mRNAs. miRNA-expression confers prognostic significance for event-free and overall survival, as do respective target-gene signatures.

**Conclusions:**

The myeloma-miRNome confers a pattern of small changes of individual miRNAs impacting on gene-expression, biological functions, and survival.

## INTRODUCTION

Multiple myeloma is a malignant disease of terminally differentiated plasma cells accumulating in the bone marrow [1]. Under the surface of a rather homogenous phenotype, myeloma is characterized by a pronounced molecular heterogeneity in terms of genetic alterations and changes of gene expression compared to normal bone marrow plasma cells [2–9]. These expression changes can be driven either directly by said genetic alterations, or indirectly by changes in signaling, e.g. due to altered external stimuli mediated by a changing microenvironment [2, 4, 6–8, 10]. Both can either act directly on gene expression or on its mediators. Prominent examples of the latter are microRNAs (miRNAs). miRNAs are non-protein-coding RNAs that function as regulators of mRNA stability and translation [11, 12]. miRNAs posttranscriptionally repress the expression of their target genes, while an up-regulation of gene expression in eukaryotes has been found under specific conditions, e.g. with specific transcripts, in distinct cell types [13]. A single miRNA is typically involved in the regulation of several hundred mRNAs. In turn, several miRNAs regulate one cognate mRNA. miRNAs are predicted to regulate more than 60% of protein coding transcripts in the human genome [14]. By this means, miRNAs participate in physiological and pathological processes, including differentiation, angiogenesis, apoptosis, development of cancer, metastasis, and drug resistance, and are described as potential diagnostic or prognostic biomarkers and therapeutic targets, e.g. in monoclonal gammopathy of unknown significance (MGUS) and multiple myeloma [11, 12, 14–23].

Referring to multiple myeloma, several global miRNA-profiling studies with impact on gene expression, biological relevance, and survival have been published, and imply a possible association with myeloma pathogenesis and molecular sub-entities in terms of specific chromosomal aberrations or gene expression-based high-risk groups [22, 24–31].

Here, we assess three main questions: i) What is the number and magnitude of changes in miRNA-expression between normal bone marrow plasma cells and myeloma- or MGUS-samples, and between MGUS and myeloma? Do these relate to single highly changing “myeloma-miRNAs”, or a network of small changes? ii) What is the biological relevance of these changes on miRNA-level and how does miRNA-expression impact on gene expression? iii) Is there a prognostic significance in multiple myeloma, and what is its background?

## RESULTS

### miRNA expression in malignant vs. normal plasma cells

Comparison of miRNA expression at genome wide level of primary myeloma cells and normal plasma cells identified 38 (6.8%) and 29 (5.2%) miRNAs to be significantly down- or up-regulated in myeloma cells compared to their normal counterpart after controlling the false-discovery-rate at a level of 5% with fold changes (FC) ranging from −3.1 (minus sign depicting down-regulation in myeloma cells) to 4.6 (Table 1). When comparing MGUS-samples to those from healthy donors, eight miRNAs (1.4%) were significantly up- and twelve (2.1%) down-regulated (Supplementary Table S3). Comparison of myeloma cells to cells from MGUS-patients revealed three differentially expressed miRNAs (0.5%), i.e. miR-200b*, miR-432 and miR-486-3p (Supplementary Table S3). No significant difference could be found between early- vs. late-stage myeloma (Durie-Salmon stage I vs. II and III).

**Table 1:** Differentially expressed miRNAs between normal plasma cells and myeloma cells

A				
miRNA	logFC	FC	Average expression	Adjusted *P*-value
miR-628-3p	1.64	3.1	10.28	< 0.001
miR-30b*	1.44	2.7	11.41	0.003
miR-490-5p	1.37	2.6	9.59	< 0.001
miR-155	1.36	2.6	9.90	0.002
miR-553	1.29	2.4	9.48	< 0.001
miR-659	1.26	2.4	11.44	< 0.001
miR-516b	1.24	2.4	9.76	< 0.001
miR-500*	1.24	2.4	10.03	< 0.001
miR-483-5p	1.15	2.2	11.56	0.003
miR-198	1.06	2.1	10.28	0.005
miR-200b*	1.05	2.1	9.86	< 0.001
miR-768-5p	0.96	1.9	9.68	0.04
miR-411	0.95	1.9	8.58	< 0.001
miR-192	0.95	1.9	9.61	0.02
miR-450a	0.91	1.9	8.24	< 0.001
miR-625	0.89	1.9	9.75	0.04
miR-500	0.84	1.8	9.50	< 0.001
miR-615-3p	0.83	1.8	10.89	0.001
miR-770-5p	0.81	1.8	8.15	0.004
miR-371-5p	0.81	1.8	11.71	0.03
miR-125b-1*	0.72	1.6	10.41	0.003
miR-645	0.71	1.6	9.10	< 0.001
miR-654-5p	0.71	1.6	9.94	0.02
miR-552	0.70	1.6	9.91	0.003
miR-28-3p	0.69	1.6	9.36	0.003
miR-7	0.66	1.6	8.88	0.04
miR-126	0.64	1.6	8.50	0.01
miR-518c*	0.63	1.5	11.38	0.02
miR-885-5p	0.63	1.5	8.84	0.01
miR-548a-5p	0.61	1.5	9.13	0.03
miR-425*	0.61	1.5	8.97	0.009
miR-27a*	0.58	1.5	8.76	0.04
miR-331-5p	0.57	1.5	8.77	0.008
miR-99b*	0.53	1.4	9.88	0.003
miR-549	0.51	1.4	8.53	0.04
miR-576-3p	0.49	1.4	8.49	0.02
miR-373*	0.47	1.4	8.49	0.03
miR-125a-3p	0.44	1.4	8.62	0.03
Differentially expressed miRNAs between normal bone marrow plasma cells and myeloma cells				
**B**				
miR-548a-3p	−0.46	−1.4	8.33	0.03
miR-200a*	−0.52	−1.4	9.60	0.02
miR-372	−0.54	−1.5	9.15	0.01
miR-148a*	−0.55	−1.5	9.15	0.05
miR-370	−0.55	−1.5	12.87	0.01
miR-29a*	−0.69	−1.6	8.78	0.04
miR-487b	−0.75	−1.7	10.41	0.02
miR-605	−0.78	−1.7	10.50	0.01
miR-634	−0.79	−1.7	10.21	0.004
let-7g	−0.85	−1.8	9.79	0.01
miR-29c*	−0.87	−1.8	9.12	0.003
miR-487a	−0.91	−1.9	10.46	0.003
miR-302b	−0.94	−1.9	10.74	< 0.001
miR-30b	−0.97	−2.0	10.70	0.007
miR-30d	−0.97	−2.0	9.85	0.004
miR-374a	−1.00	−2.0	9.22	0.009
let-7i	−1.03	−2.0	10.14	0.002
miR-148b	−1.07	−2.1	10.62	0.03
miR-30a	−1.11	−2.2	10.51	0.03
miR-195	−1.14	−2.2	10.35	0.03
miR-519d	−1.15	−2.2	11.70	0.004
miR-24	−1.22	−2.3	10.32	0.02
let-7f	−1.24	−2.4	10.02	< 0.001
miR-26a	−1.36	−2.6	11.13	0.02
miR-148a	−1.51	−2.8	12.30	0.01
miR-29b	−1.89	−3.7	12.95	0.002
miR-29c	−1.93	−3.8	12.14	0.001
miR-29a	−2.17	−4.5	12.48	< 0.001
miR-142-3p	−2.20	−4.6	12.05	0.007

**A.** 38 miRNAs are significantly down-regulated, **B.** 29 miRNAs are up-regulated in myeloma cells compared to their normal counterpart. miRNAs are listed according to the height of their (log) fold change (FC). Minus sign depicting lower expression in normal plasma cells and thus an up-regulation in myeloma cells.

To appraise the order of magnitude of the differences in miRNA-expression, we compared miRNA-expression of normal, MGUS-samples and primary myeloma cells each with myeloma cell lines. In this case, 89 (15.9%), 173 (30.9%), and 410 (73.3%) miRNAs were found to be differentially expressed with FC of 7.2 to −6.2, 5.7 to −5.5, and 7.4 to −4, respectively, i.e. comparably higher compared to the differences observed within primary samples. Taking all four aforementioned sample types together, miR-302b, miR-490-5p, and miR-155 were concomitantly differentially expressed between all. The latter two are significantly lower expressed in cells from MGUS- and myeloma patients compared to their normal counterpart, while miR-302b was significantly higher (Table 1, Supplementary Figure S1, Supplementary Table S3).

**Figure 1:**
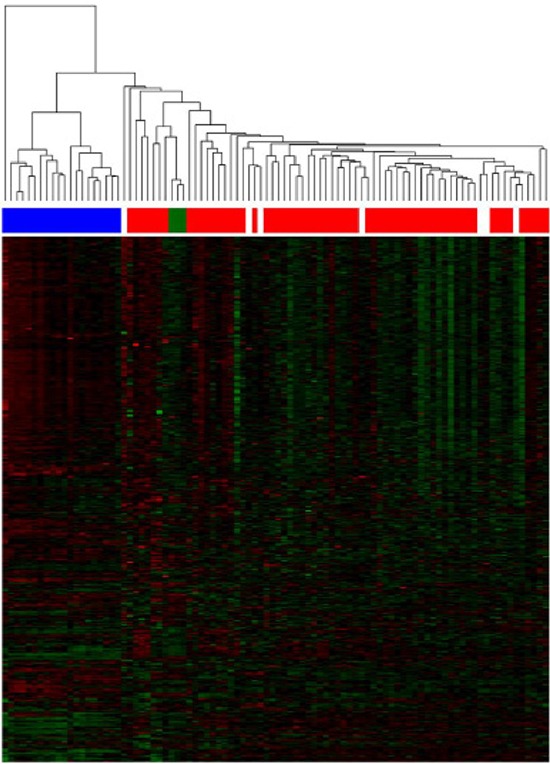
Unsupervised clustering based on miRNA expression The unsupervised clustering shows normal bone marrow plasma cells (depicted in green) clustering together in a sub-branch within the myeloma cell samples (depicted in red). Patients with MGUS (depicted in white) disperse over the normal/malignant plasma cell cluster, no sub-clusters can be found. Human myeloma cell lines (depicted in blue) clustering together in a separate branch.

In the unsupervised hierarchical clustering, normal and malignant plasma cells cluster in one branch, myeloma cell lines aggregate together in a separate one (Figure 1, Supplementary Figure S2). If MGUS-samples are included, they disperse over the normal/malignant plasma cell cluster. But for normal plasma cell samples, no sub-clusters can be identified.

### Biological relevance of miRNA expression

To analyze whether the comparably small changes in normal vs. malignant plasma cells and within the latter have biological significance, we investigated differences in terms of associations with i) biological variables, ii) chromosomal aberrations, and iii) gene expression-based high-risk scores.

#### Proliferation

Ten miRNAs showed a significant association with the gene expression-based proliferation index (GPI; Supplementary Table S4). The latter is a gene expression-based measure of myeloma cell proliferation based on genes over-expressed in proliferating malignant (i.e. myeloma cell lines) as well as non-malignant cells (i.e. polyclonal plasmablasts) compared to non-proliferating, non-malignant cells (i.e. normal bone marrow plasma cells, memory B-cells) [3]. Of the 10 miRNAs, three were negatively correlated with the GPI as continuous variable, while seven positively (Supplementary Table S5). Of the latter, five belong to the miR-17–92 cluster. Between GPI^low^ and GPI^high^, eight miRNAs were differentially expressed. All of them were up-regulated in the GPI^high^-group with a maximal FC of 2.3 (Supplementary Table S6), six miRNAs belonging to the miR-17–92 cluster.

#### Tumor mass

Two miRNAs, miR-135a and miR-596, show a significant association with the International Staging System (ISS) (*P* = .05; *P* = .02) (Table 2) as surrogate of tumor mass. In contrast, there was no correlation of miRNA expression with β2-microglobulin alone.

**Table 2:** Association with survival, risk scores and cytogenetic aberrations

miRNA	Expression cut	EFS	OS	GPIC		GPI	IFM score	UAMS score	t(4;14)	t(11;14)	del17p	gain 1q21	del13q	ISS
		*P*-value	*P*-value	*P*-value	mean GPI	*P*-value	*P*-value	*P*-value	*P*-value	*P*-value	*P*-value	*P*-value	*P*-value	*P*-value
miR-135a	8.38	**0.004**	**0.03**	0.3	189 / 215	0.8	**0.02**	0.06	0.06	0.7	0.7	0.3	0.2	**0.05**
miR-135b	8.18	**< 0.001**	**< 0.001**	0.08	178 / 215	0.7	**0.03**	**0.04**	**0.01**	0.7	1	0.4	0.4	0.4
miR-200a	8.42	**0.01**	**0.02**	**0.01**	180 / 245	0.06	**0.006**	0.2	0.4	0.07	0.4	1	1	0.2
miR-200b	8.89	**0.03**	0.8	0.3	184 / 208	0.7	0.5	1	0.7	0.7	0.7	1	0.4	0.2
miR-596	9.61	**0.02**	**0.001**	**0.02**	174 / 225	**0.04**	0.1	**< 0.001**	**0.001**	0.1	0.2	0.4	**0.07**	**0.02**

Shown is the association of the five survival relevant miRNAs with event-free (EFS) and overall survival (OS), as well as the IFM- and UAMS gene expression-based risk scores and cytogenetic aberrations. Significant values are depicted in bold. GPI(C), gene expression-based proliferation index (as continuous variable), the mean of the low and high GPI group is shown for GPIC; IFM score, risk score of the Intergroupe Francophone du Myélome published by Decaux et al.; UAMS score, risk score of the University of Arkansas for Medical Sciences published by Shaughnessy et al.; ISS, International staging system.

#### Chromosomal aberrations

Regarding translocation t(4;14), three miRNAs are differentially expressed, i.e. miR-135a, miR-596, and miR-432*, being significantly down-regulated when a t(4;14) is present with a maximal FC of 1.9 (Supplementary Table S6B). In samples with t(11;14) vs. none, 24 miRNAs were significantly differentially expressed. Fourteen miRNAs were up-regulated, ten down-regulated (FC 2.04 to −1.92) (Supplementary Table S6B). Regarding gain of 1q21 vs. no gain, miR-501-3p is significantly up-regulated (FC 1.62). No association was found for deletion 17p13. For deletion 13q14, three miRNAs are significantly differentially expressed, namely miR-23a, miR-23b, and miR-767-5p. The latter is up-regulated in samples from patients without this aberration (FC 1.37), the other two are down-regulated (FC 1.39 and 1.40, respectively) (Supplementary Table S6B). In hyperdiploid patients a significant down-regulation of miR-21, miR-22, miR-93, miR-125b, and miR-374b with a maximal FC of 2.1 is present (Supplementary Table S6B).

#### Gene expression-based high-risk scores

Four miRNAs are significantly positively associated and correlate with the University of Medical Sciences (UAMS) 70-gene risk-score, i.e. miR-135b, miR-432*, miR-583, and miR-596 (Supplementary Table S4, S5). The same are also differentially expressed between low-risk vs. high-risk with a maximal FC of 1.8 (Supplementary Table S6A), all being up-regulated in high-risk patients. Regarding the Intergroupe Francophone du Myélome (IFM) 15-gene score, 32 miRNAs are significantly associated with, i.e. 24 showed a negative association, eight a positive one (Supplementary Table S4). Thirty-nine miRNAs show a significant correlation with the IFM-score as continuous variable: ten miRNAs are negatively correlated, 29 positively (Supplementary Table S5). Of the latter, nine miRNAs belong to the miR-17–92 cluster. Comparing low-risk vs. high-risk samples, 34 miRNAs are differentially expressed (Supplementary Table S6A). Of these, 10 are significantly up-, 24 down-regulated in low-risk patients with nine miRNAs belonging to the miR-17–92 cluster (maximal FC: 1.42 to −1.74). In contrast to gene expression-based high-risk scores we could not find a correlation of miRNA expression with molecular classifications of multiple myeloma.

#### Correlation of miRNA- and mRNA-expression

Twelve of 559 miRNAs investigated are significantly correlated with the expression of 24 mRNAs with *r* ≥ 0.6 or *r* ≤-0.6 (Table 3). Seventeen correlations were negative, twelve positive, and five miRNAs (miR-19b, miR-103, miR-106b, miR-424, and miR-623) were correlated with more than one mRNA. miR-103, miR-106b, and miR-424 were significantly up-regulated in GPI^high^-patients and IFM high-risk (Supplementary Table S6A). Twenty-two of these 24 mRNAs showed a prognostic impact and were able to significantly delineate two groups of patients with different event-free (EFS) and/or overall survival (OS) (Table 3, Supplementary Table S7).

**Table 3:** Correlation between miRNA and mRNA

Gene symbol	mRNA probeset	miRNA	Correlation (r)	adjusted *P*-value	EFS				OS			
					Cut-off	HR	[95% CI]	adj. *P*-value	Cut-off	HR	[95% CI]	adj. *P*-value
*KCTD11*	235857_at	let-7f	0.60	0.02	4.81	0.803	[0.567;1.138]	0.2	4.35	0.65	[0.38;1.113]	0.1
*EXTL2*	209537_at	let-7g	−0.60	0.02	8.62	0.708	[0.455;1.101]	0.1	7.32	1.478	[0.933;2.342]	0.1
*XRCC4*	205071_x_at	let-7i	−0.62	0.02	8.69	0.638	[0.438;0.928]	0.02	8.86	0.534	[0.295;0.967]	0.04
*ITPR1*	203710_at	miR-19b	0.60	0.02	5.17	1.479	[1.035;2.115]	0.03	3.59	2.433	[1.119;5.289]	0.03
*SELM*	226051_at	miR-19b	−0.62	0.02	11.09	0.742	[0.527;1.045]	0.09	9.58	0.463	[0.294;0.732]	0.001
*GRN*	200678_x_at	miR-100	−0.61	0.02	12.48	0.55	[0.335;0.901]	0.02	12.46	0.462	[0.223;0.96]	0.04
*SIN3A*	225135_at	miR-103	0.60	0.02	5.75	1.896	[1.367;2.631]	< 0.001	5.81	2.097	[1.351;3.254]	0.001
*GRN*	200678_x_at	miR-103	−0.62	0.02	12.48	0.55	[0.335;0.901]	0.02	12.46	0.462	[0.223;0.96]	0.04
*SELM*	226051_at	miR-103	−0.62	0.02	11.09	0.742	[0.527;1.045]	0.09	9.58	0.463	[0.294;0.732]	0.001
*FBXO11*	219208_at	miR-106b	0.72	< 0.001	6.37	1.634	[1.148;2.325]	0.006	6.39	2.124	[1.355;3.329]	0.001
*TMPO*	209754_s_at	miR-106b	0.66	0.005	6.43	1.587	[1.119;2.252]	0.01	4.34	1.826	[1.107;3.01]	0.02
*BUB1*	209642_at	miR-106b	0.64	0.01	3.65	2.368	[1.68;3.338]	< 0.001	4.37	2.965	[1.894;4.64]	< 0.001
*MCM4*	222036_s_at	miR-106b	0.63	0.01	6.89	1.883	[1.358;2.612]	< 0.001	7.42	2.545	[1.64;3.947]	< 0.001
*SIN3A*	225135_at	miR-106b	0.63	0.01	5.75	1.896	[1.367;2.631]	< 0.001	5.81	2.097	[1.351;3.254]	0.001
*RPS6KA3*	203843_at	miR-106b	0.62	0.02	7.39	1.699	[1.186;2.433]	0.004	7.39	3.083	[1.971;4.821]	< 0.001
*BUB1B*	203755_at	miR-106b	0.60	0.02	7.24	1.969	[1.403;2.764]	< 0.001	8.61	2.951	[1.725;5.05]	< 0.001
*GRN*	200678_x_at	miR-106b	−0.61	0.02	12.48	0.55	[0.335;0.901]	0.02	12.46	0.462	[0.223;0.96]	0.04
*HIST1H2AC*	215071_s_at	miR-186	−0.72	< 0.001	11.59	1.431	[1.011;2.026]	0.04	11.85	1.889	[1.176;3.036]	0.009
*HIST1H2AC*	215071_s_at	miR-374a	−0.68	0.002	11.59	1.431	[1.011;2.026]	0.04	11.85	1.889	[1.176;3.036]	0.009
*TNIP2*	232160_s_at	miR-424	0.70	< 0.001	6.06	1.898	[1.304;2.762]	0.001	6.61	1.956	[1.261;3.035]	0.003
*GINS2*	221521_s_at	miR-424	0.65	0.01	6.65	2.223	[1.596;3.096]	< 0.001	6.93	2.532	[1.627;3.939]	< 0.001
*EPB41L2*	201719_s_at	miR-602	−0.62	0.02	5.60	0.672	[0.482;0.935]	0.02	5.68	0.512	[0.319;0.819]	0.005
*WEE1*	212533_at	miR-623	−0.61	0.02	8.59	1.834	[1.321;2.546]	< 0.001	9.53	2.32	[1.484;3.629]	< 0.001
*GCLM*	236140_at	miR-623	−0.62	0.02	5.72	1.563	[1.118;2.185]	0.009	5.56	1.503	[0.964;2.344]	0.072
*MCM6*	201930_at	miR-623	−0.62	0.02	8.30	2.169	[1.269;3.705]	0.005	10.75	2.705	[1.63;4.491]	< 0.001
*STRN*	236388_at	miR-623	−0.63	0.01	6.39	1.901	[1.297;2.788]	0.001	6.09	1.98	[1.239;3.163]	0.004
*RAPH1*	225188_at	miR-623	−0.63	0.01	5.65	1.824	[1.315;2.531]	< 0.001	6.10	1.641	[1.058;2.545]	0.03
*ACTR2*	200729_s_at	miR-623	−0.64	0.01	11.79	1.656	[1.167;2.349]	0.005	12.04	2.521	[1.515;4.193]	< 0.001
*RMI2*	226456_at	miR-623	−0.66	0.005	4.85	1.816	[1.296;2.543]	0.001	7.08	2.927	[1.689;5.073]	< 0.001

Shown are mRNAs that correlate with miRNAs with a correlation coefficient *r* ≥ 0.6 or *r* ≤ −0.6 and their impact on event-free (EFS) and overall survival (OS) using the indicated cut-offs.

HR, hazard ratio; CI, confidence intervall.

The three miRNAs differentially expressed between t(4;14) vs. none regulate 379 genes, 355 of which are represented on the microarray. Of these, 43 are significantly differentially expressed between the two entities with 23 being up-regulated. For the eight miRNAs differentially expressed between GPI^high^ vs. GPI^low^ regulating 1693 genes (1586 represented on the DNA-microarray), 410 are differentially expressed (362 up-regulated).

### Prognostic significance of miRNA-expression

**Figure 2:**
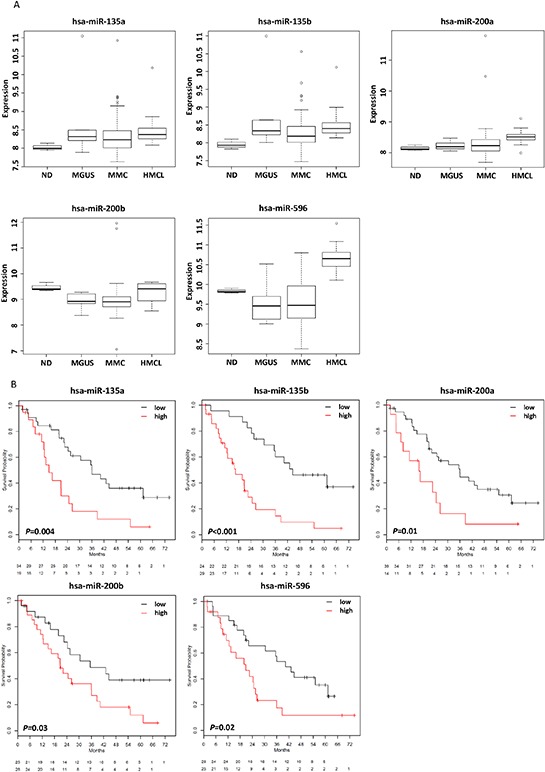

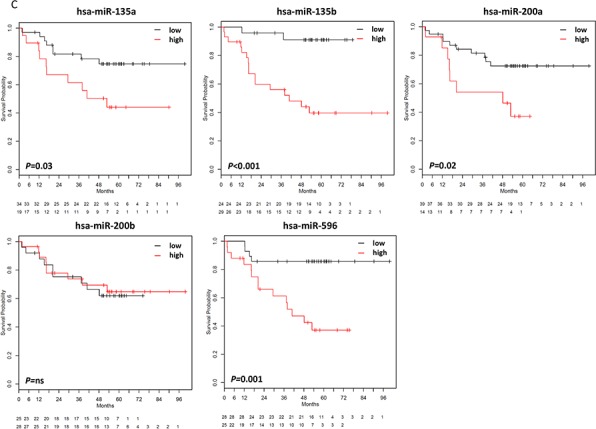
miRNAs associated with survival **A.** Expression of five miRNAs was associated with event-free and all but one with overall survival and allowed the delineation of prognostic groups. These miRNAs are associated with significantly different **B.** event-free and **C.** overall survival (the latter but for miR-200b). Overall survival. Patients with low expression of these miRNAs show a favorable prognosis. hsa, Homo sapiens.

We next assessed the prognostic relevance and without correction for multiple testing finding 72 miRNAs to be significantly associated with EFS, 69 with OS. Corrected for multiple testing, five miRNAs remained significantly associated with EFS as continuous variable, i.e. miR-135a, miR-135b, miR-200a, miR-200b, and miR-596, and, but for miR-200b, also with OS (Figure 2). For all miRNAs, a high expression delineates a group with inferior EFS and OS.

#### miRNA signature

**Figure 3:**
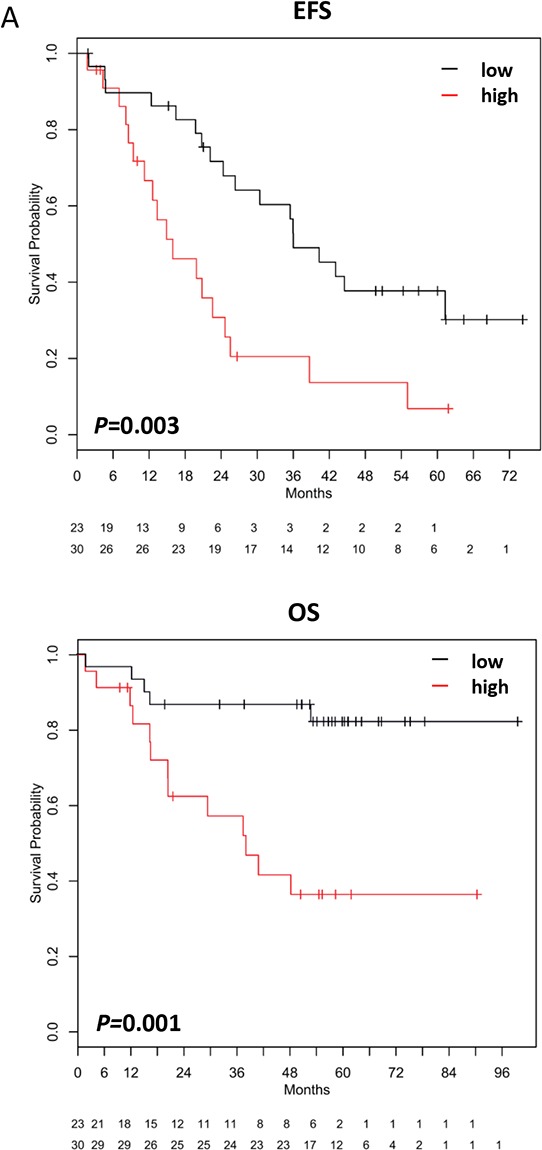

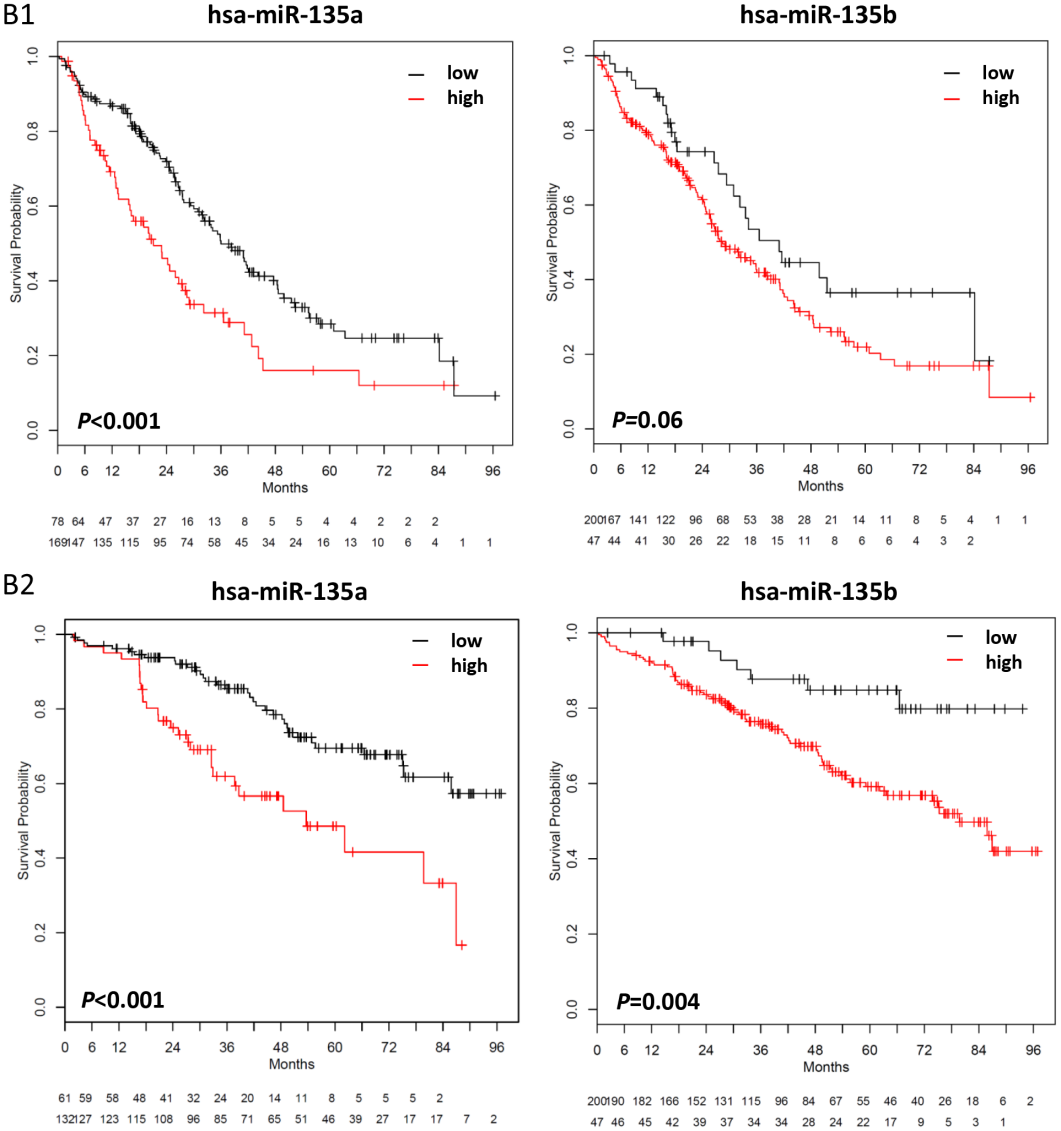

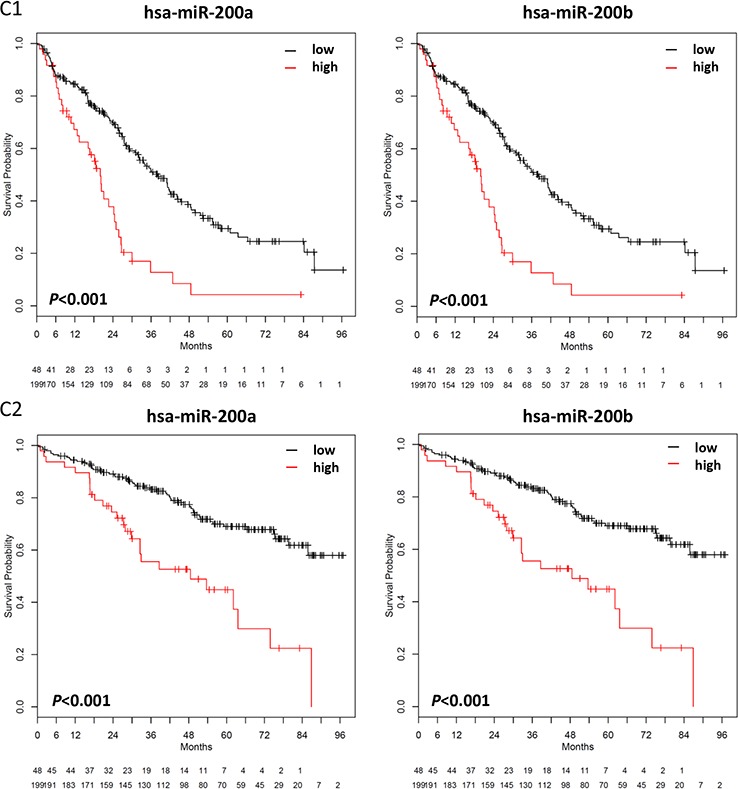
miRNA- and target gene signatures **A.** A miRNA-signature for survival prediction was constructed using principal component analysis. This signature significantly predicts for event-free (EFS; *P* =.003) and overall survival (OS; *P* =.001), with a low risk-score delineating a group of patients with inferior survival. **B, C.** By using miRWalk, predicted target genes for miR-135a, miR-135b, miR-200a, and miR-200b are significantly associated with (B1,C1) event-free survival (miR-135a, *P* <.001; miR-135b, *P* = ns (.06); miR-200a, *P* < .001; miR-200b, *P* < .001) and (B2,C2) overall survival (miR-135a, *P* < .001; miR-135b, *P* =.004; miR-200a, *P* < .001; miR-200b, *P* < .001). For validation of the data on an independent cohort of patients treated within the total therapy 2 protocol [53], please see Supplementary Figure S4.

A miRNA signature for survival prediction in myeloma patients based on the five survival relevant miRNAs could be constructed delineating two groups of patients with significantly different EFS (*P* = .003) and OS (*P* = .001) (Figure 3A).

### Background of prognostic relevance of miRNAs

#### Proliferation

Of the five survival relevant miRNAs, the GPI as continuous variable is significantly higher in patients with a high expression of miR-596 (*P* = .02), and miR-200a (*P* = .01) (Table 2). Regarding miRNA-596, all patients classified as GPI^high^ showed a significantly higher expression (*P* = .04).

#### Chromosomal aberrations

Expression of several survival relevant miRNAs is associated with the presence of specific chromosomal aberrations (Table 2). High expression of miR-135a (*P* = .02), miR-135b (*P* = .02), and miR-596 (*P* = .003) is significantly associated with a t(4;14). Besides, miR-135a (*P* = .01) and miR-596 (*P* = .004) are differentially expressed between t(4;14) vs. none with an up-regulation in myeloma cells from patients with this translocation. Furthermore, a high miR-596 expression is associated with deletion 13q14 (*P* =.04). No associations was found for gain of 1q21, deletion 17p13, or t(11;14), albeit a tendency for the latter regarding miR-200a (*P* =.07).

#### Gene expression-based high-risk scores

The IFM-score is significantly associated with miR-135a (*P* =.02), miR-135b (*P* =.03), and miR-200a (*P* =.006). Samples with a high miR-135b-expression were significantly more frequently classified as being high-risk. For the UAMS-score, miR-135b (*P* =.04) and miR-596 (*P* < .001) were significantly associated with (Table 2). Samples with a high expression of these miRNAs are significantly more often classified as high-risk, and both are also significantly differentially expressed between low- and high-risk patients with an up-regulation in the latter (*P* =.03; *P* =.002).

#### Tumor mass

Both miRNAs which were significantly associated with the ISS-stage as a surrogate of tumor mass (see above), i.e. miR-135a (*P* = .05) and miR-596 (*P* = .02) (Table 2), are also significantly prognostic regarding EFS and OS. Patients with a high expression of miR-596 show a significantly higher stage (ISS stage III).

#### Impact of prognostic miRNAs on gene expression

We next investigated whether the predicted target genes are likewise survival relevant. Indeed, a significant association could be found for EFS and OS (Supplementary Figure S3). Target gene signatures for miR-135a, miR-135b, miR-200a, and miR-200b significantly predict for EFS and OS with a high “target risk-score” delineating a group with inferior survival (Figure 3B, 3C). This was validated on an independent cohort of 345 myeloma patients treated within the total therapy 2 trial (Supplementary Figure S4). Patients with a favorable score are more frequently UAMS low-risk and have a lower GPI. In an ANOVA-model including the target risk-scores of miR-135a, miR-135b, miR-200a, miR-200b and the UAMS-score, the score of miR-200a remains significant (*P* =.04) alongside the UAMS-score (*P* =.02). Regarding the IFM-score, target risk-scores of miR-135a and miR200a (*P* <.001; *P* =.04) unlike the IFM-score show significance. In an ANOVA-model including target risk-scores and GPI as continuous variable, only the latter remains significant (*P* <.001).

## DISCUSSION

### miRNA expression in normal and malignant plasma cells

The miRNome of malignant plasma cells significantly differs from its normal counterpart in 67 of 559 miRNAs tested (12%) with FC of 4.6 to −3.1, strong enough to drive its own normal plasma cell sub-cluster within primary myeloma samples. Comparing normal plasma cells with those from MGUS-patients, 20 miRNAs are differentially expressed, 18 of which (90%) are likewise differentially expressed compared to myeloma cells. Between MGUS- and myeloma patients, only 3 significantly different miRNAs could be found. On miRNA-level, plasma cells from MGUS-patients thus seem to resemble myeloma rather than normal plasma cells. This could either suggest a role of these miRNAs early in the development of MGUS and subsequently myeloma, or an early impact of pathogenetic changes on miRNA-expression.

miRNAs have been published to be globally down-regulated in cancer acting as negative regulators of tumorigenesis [32–34], others showed an up-regulation in tumor samples or those from high-risk patients [27, 30, 35]. We found a comparable number being up- or downregulated in normal compared to malignant plasma cells, thus a network of small bidirectional changes, but not a single or group of “unifyingly”, differentially expressed miRNAs.

Taken together, we find a network of small changes comparing malignant to normal plasma cells, not highly changing “myeloma-miRNAs”, with MGUS being closely related to myeloma. Cell lines show a much higher difference compared to both samples types.

### Biological relevance of miRNA-expression

We next investigated whether these rather small changes show an association with biological variables, i.e. proliferation, tumor mass, chromosomal aberrations, and gene expression-based risk scores, and subsequently, whether miRNA-expression impacts on gene expression.

#### Proliferation

Ten miRNAs showed a significant association with the GPI, eight were differentially expressed between GPI^low^ vs. GPI^high^. All of them were up-regulated in the GPI^high^-group and six belong to the miR-17–92 cluster. Of these, miR-106b correlates with *BUB1* (see subsequent paragraph) being part of the expression-based proliferation index by Zhan *et al*. [7]. The miR-17–92 cluster was described as potential oncogene (onco-miR-1) targeting pro-apoptotic genes [27, 36]. Up-regulation in GPI^high^-patients could thus contribute to anti-apoptotic signaling in this group, in agreement with members of the miR-17–92 cluster being within the most up-regulated miRNAs in proliferating myeloma cell lines compared to (non-proliferating) normal and malignant plasma cells. This association with proliferation is in line with the rather large changes between proliferating myeloma cell lines and the other samples types. This is also in accordance with data from Pichiorri *et al*. who showed members of the oncogenic miR-17–92 and miR-106b-25 clusters, both sharing a high degree of homology, being upregulated in malignant vs. normal plasma cells with the highest fold changes in myeloma cell lines [27]. Interestingly, a high expression of members of the miR-17–92 cluster is associated with a shorter progression-free survival in myeloma patients [37].

#### Tumor mass

ISS-stage as surrogate of tumor mass is associated with the expression miR-135a and miR-596. For the latter, patients with a high expression show a significantly higher ISS-stage. Interestingly, both miRNAs were also prognostic regarding EFS and OS (see below).

#### Chromosomal aberrations

miRNAs have been shown to be associated with different cytogenetic subgroups of myeloma patients [25, 26, 29, 31]. We likewise found an association with the IgH-translocations t(4;14) and t(11;14), as well as del13q14, and 1q21-gain, but the overlap to previous studies is limited. Besides the limitations discussed below, this could be explained in part by the fact that in these studies myeloma samples with a particular aberration have been compared with normal plasma cells [25] instead of patient samples without this aberration as in our study.

#### Gene expression-based high-risk scores

For both, the UAMS- [38] and the IFM-score [39], we identified a significant association with miRNA-expression. All four miRNAs being significantly positively correlated with the UAMS-score are up-regulated in high-risk patients. Nevertheless, we found no overlap with the work by Zhou *et al*. [30], probably due to the limitations discussed below. However, 6 of 8 miRNAs which are significantly higher expressed in GPI^high^-patients, and five of ten miRNAs being positively correlated with the GPI are overlapping, but for miR-103 all being part of the miR-17–92 cluster. Among others, miRNAs of the miR-17–92 and miR-106b-25 cluster are also positively correlated with the IFM-score being up-regulated in high-risk patients and have been described being upregulated in malignant vs. normal plasma cells with the highest FC in cell lines [27].

#### Impact on gene expression

As about 60% of the human transcriptome is regulated by miRNAs [14], we investigated if there is a correlation between miRNA- and mRNA-expression. We found 12/559 miRNAs to be correlated with 24 genes with *r* ≥ 0.6 or *r* ≤ −0.6. Twenty-two genes were able to delineate two groups of patients with significantly different EFS and/or OS. miR-19b and miR-106b are members of the miR-17–92 cluster and correlated to genes, e.g. *BUB1* and *BUB1B*, that have been described as components of the mitotic checkpoint control [40]. miR-106b is also correlated to *TMPO* as part of the UAMS 70-gene risk-score [38]. A differential expression regarding predicted target genes of differentially expressed miRNAs can likewise be found for biological features, e.g. t(4;14) vs. none, or GPI^high^ vs. GPI^low^.

Our findings need to be interpreted on the background of general miRNA-regulatory mechanisms: A single miRNA is typically involved in the regulation of hundreds of mRNAs, and several miRNAs regulate one cognate mRNA. Direct target genes can in turn impact on several other (indirect target) genes or miRNAs [41]. Single miRNA-target interactions typically yield less than 2-fold reductions in protein expression [42]. To yield observable biological effects, seemingly all members of seed and non-seed families with overlapping functions need to be knocked-out [43]. The more striking is that the small differences in miRNA-expression, indeed associate with biological variables, and impact on gene expression.

### Prognostic impact of miRNA expression and its background

Given their biological significance, we investigated whether this transmits into and/or parallels survival relevance. Indeed, a comparably high number of miRNAs was significantly associated with survival. Those remaining significant after correction for multiple testing, i.e. miR-135a, miR-135b, miR-200a, miR-200b, and miR-596, have recently been published to play a role in solid tumors and hematologic cancers, respectively, e.g. functioning as either oncogenes or tumor-suppressors and prognostic markers [44–48]. In myeloma, a high expression of these miRNAs delineates a group of patients with inferior survival. The same holds true if a miRNA-signature is build based on these miRNAs. Associations with proliferation, chromosomal aberrations, and gene expression-based high-risk could be possible explanations. Indeed, a high expression of miR-135a, miR-135b, and miR-596 is significantly associated with a t(4;14), miR-596 also with del13q14. Although patients with a high expression of miR-596 and miR-200a show a significantly higher GPI which is in turn associated with 1q21-gain [3], no association with this aberration could be found. Except for miR-200b, all survival relevant miRNAs show also a significant association with gene expression-based risk-scores [38, 39]. For miRNA-135b and miR-596, samples with a high expression were significantly more frequent in the particular high-risk group. As all GPI^high^-patients showed a high miR-596-expression, miR-596 is not only linked to survival and high-risk disease in terms of chromosomal aberrations, tumor mass, and the UAMS-score [38], but also to proliferation, one of the strongest independent risk-factors in myeloma [3]. Besides these associations, published data also suggest a possible role in angiogenesis as shown for exosomal miR-135b derived from myeloma cells which enhances angiogenesis by targeting factor-inhibiting HIF-1 [49], or myeloma bone disease, with miR-135b, being upregulated in mesenchymal stromal cells from myeloma patients, being involved in an impaired osteogenic differentiation process [21]. Next, we investigated whether the signature of regulated genes impacts on survival, which is the case (Figure 3B,C).

Taken together, differences in miRNA-expression between normal and malignant plasma cells are small, but biologically relevant with impact on survival.

### Caveats

Compared to gene expression profiling, there are few whole miRNA-profiling studies in myeloma, with comparatively small patient cohorts, and varying results. The latter is especially true if the lack of overlap between different studies is considered [22, 24–28, 30]. Reasons can be seen in technical and biological imponderabilities. First, the limited representativeness of small cohorts due to the significantly lower availability of samples for miRNA- compared to mRNA-profiling because of the comparatively high cell number necessary to obtain sufficient miRNA. This will likely be overcome by next generation sequencing approaches necessitating lower amounts of starting material. Second, miRNAs show relatively small FC compared to mRNAs regarding differential expression between normal and malignant plasma cells. Correcting for multiple testing to reduce false discoveries thereby takes a higher toll. Identified miRNAs are thus likely “truly” differentially expressed, whereas those not identified are not necessarily not differentially expressed. In this case, small differences, also in the cohort constitution, might lead to the selection of different miRNAs in different studies. The same holds true for different normalization strategies [50]. The differences between studies are in turn in line with our interpretation of the miRNome in myeloma as a complex network with small changes in individual miRNAs and absence of single highly changing “unifying” miRNAs. The latter would presumably have been identified in the majority of studies. Therefore, current functional validation strategies down- or up-regulating single miRNAs by several log-decades can thus only with great care be taken as validation of the small changes of a comparably large number of miRNAs. On the basis of the said above, it currently seems difficult to exploit the miRNome in a diagnostic or therapeutic manner.

In conclusion, miRNAs form a network in which small changes in individual miRNAs act together in changing the (global) miRNA- and mRNA-expression pattern. The miRNome is thus a complex and survival relevant regulator of gene expression whose alterations have biological and prognostic impact in multiple myeloma.

## MATERIALS AND METHODS

### Patients, healthy donors and samples

Patients presenting with previously untreated multiple myeloma or MGUS at Heidelberg University Hospital, and healthy donors were included after written informed consent in the study approved by the institutional ethics committee (#229/2003 and S-152/2010). Normal and malignant plasma cells were purified as published [2–6]. Median plasma cell purity after CD138-sorting was 96% [range: 80–100%] as assessed by flow cytometry. Myeloma cell lines were purchased from the German Collection of Microorganisms and Cell Cultures (Braunschweig, Germany) or American Type Culture Collection (Wesel, Germany), HG-1 was generated in the Myeloma Research Laboratory Heidelberg, the XG-lines were generated as published [51, 52]. For an overview, see Supplementary Table S1, S2.

#### Samples for miRNA-profiling

For miRNA-profiling, 62 primary myeloma cell-samples, 7 MGUS-, 3 samples from healthy donors (pooled from three donors each), and 20 myeloma cell lines were investigated. Of the former, 53 patients underwent frontline high-dose chemotherapy with 200mg/m^2^ melphalan and autologous stem cell transplantation (Supplementary Table S1, S2).

#### Samples for gene expression profiling

Three-hundred-thirty-two primary myeloma cell samples, 22 MGUS-, 10 samples from normal donors, and 32 myeloma cell lines were used. Of these, 247 myeloma patients underwent up-front high-dose chemotherapy with 200mg/m^2^ melphalan and autologous stem cell transplantation and were available for survival analysis. As validation, an independent cohort of 345 patients treated within the total therapy 2 protocol was used [53] totaling 677 myeloma cell samples with gene expression data from which survival data were available for a total of 592 patients (Supplementary Table S1, S2).

### RNA and miRNA extraction

RNA extraction was performed using the AllPrep DNA/RNA Mini Kit (Qiagen, Hilden, Germany). The flow-through containing miRNAs was processed using the RNeasy MinElute Cleanup Kit (Qiagen). RNA quality was assessed using an Agilent 2100 Bioanalyzer (Agilent Technologies, Boeblingen, Germany).

### miRNA profiling

miRNA profiling by miChip (Exiqon LNA Array probes V9.2, Vedbaek, Denmark) was performed as published [54]. Data were deposited in the ArrayExpress repository under the accession number E-MTAB-1363. Expression of three survival relevant miRNAs (miR-135a, miR-135b, and miR-596) was validated in ten cell lines and nine primary myeloma cell samples using qRT-PCR (miQPCR). For miQPCR, RNA was extracted from 2.5 × 10^5^ cells using the Allprep-/RNeasy MinElute Cleanup Kit in 50μl RNase-free water, speedvaced and re-eluted in 10μl RNase-free water. One μl of RNA was reverse transcribed and 2.5 μl of cDNA were used for RT-PCR. cDNAs were amplified by using gene specific primers and SYBR Green (Applied Biosystems) and run on a ABI 7500 instrument (95°C for 10 min, followed by 40 cycles of 95°C for 15 sec, 60°C for 40 sec) with a dissociation step (ramping from 60°C to 95°C) as published [55]. All reactions were run in triplicates and two independent experiments.

### Gene expression profiling

Gene expression profiling was performed as published [2–4, 6] using Affymetrix U133 2.0 plus microarrays (Affymetrix, Santa Clara, CA, USA). Expression data are deposited in ArrayExpress under the accession number E-MTAB-317.

### Interphase fluorescence in situ hybridization

Analyses were performed on CD138-purified plasma cells as described [9, 56]. For an overview of used probes (Kreatech Diagnostics, Amsterdam, The Netherlands; MetaSystems, Altlussheim, Germany), see Supplementary Table S1.

### Statistical analysis

Gene expression data were assessed as previously described [2–6]. Signal calls on the miChip were preprocessed using MiChip package selecting only spots associated with human miRNAs for further preprocessing (http://www.bioconductor.org/packages/devel/bioc/html/MiChip.html). The summarized signal calls on the miChip were log2 transformed after scaling with a constant (114) so that the smallest value was 1 before log transformation. For normalization, an invariant-based normalization method was applied [50]. For unsupervised analysis, hierarchical clustering using the average linkage method with the centered Pearson correlation method was used. Differential miRNA expression was assessed using empirical Bayes statistics in linear models for microarray data [57] and *P*-values were adjusted for multiple testing controlling the false-discovery-rate as defined by Benjamini and Hochberg at a level of 5% [58]. To assess the association of total miRNA expression and miRNA target genes, respectively, with EFS and OS [3], the gene expression-based proliferation index (GPI) [3], as well as the IFM- [39] and the UAMS [38] high-risk score, Goeman's global test was applied [59]. Cox regression was used to identify miRNAs associated with EFS and OS at a significance level of *P* <.05. Maximally selected rank statistics (http://cran.r-project.org/web/packages/maxstat/index.html) were conducted to find optimal cutoffs for defining high- and low-risk groups and survival outcomes were compared using the log-rank test. Differences in clinical parameters between defined groups were investigated by Wilcoxon rank-sum test. Correlation was assessed using Pearson's correlation coefficient and the relationship between categorical variables by Fisher's Exact Test. An effect was considered as statistically significant if the *P*-value of its corresponding statistical test was ≤ 5%.

#### miRNA target prediction

Target gene prediction was carried out using miRWalk [60] integrating the predicted target transcripts of the following prediction tools: DIANA-microT, miRanda, miRWalk, miRDB, PITA, RNAhybrid and TargetScan. To limit the number of false-positives, we only retained those transcripts for further analysis identified by at least 6 out of 7 target prediction algorithms.

#### miRNA signature

A miRNA signature for survival prediction was constructed using principal component analysis on the 53 samples for which survival data were available and 5 survival associated miRNAs. First, taking the first and second principal component, two weighted averages for each sample were computed. Second, multivariate proportional hazards coefficients for the two weighted averages were assessed for EFS an OS each. Third, the score for each sample was computed as the vector product of the mean proportional hazard coefficients for EFS and OS and the samples weighted averages. Fourth, maximal selected rank statistics were used to find within the univariate score an optimal cut-off for defining a high- and low-risk group.

#### miRNA target scores

miRNA target scores were generated for the survival relevant miRNAs using 247 mRNA expression samples for which survival data were available. Each target score was generated using the predicted target genes for the specific miRNA and applying the same principal component analysis-based algorithm that was used for generating the miRNA signature (see preceding paragraph). Generated target scores were validated regarding their impact on EFS and OS using an independent cohort of 345 samples from myeloma patients treated with up-front high-dose chemotherapy and autologous stem cell transplantation within the total therapy 2 protocol [53]. First, each sample was normalized by applying preprocessing information from test group by using a documentation-by-value strategy [61]. Second, the cut-off value from the test group was used to depict the high- and low-risk group.

#### Correlation of miRNA expression with mRNA expression

For 56 samples for which miRNA- and mRNA expression data were available, Pearson correlation was calculated. The latter was used as straightforward method to analyze the relationship between miRNAs and mRNAs [62]. miRNA and mRNA expression data were filtered by 80% least variable probesets resulting in 112 miRNAs and 8088 mRNA probesets. *P*-values were adjusted for multiple testing controlling the false-discovery-rate as defined by Benjamini and Hochberg at a level of 5% [58]. Correlation cut-offs were set to *r* > 0.6 and *r* < −0.6, and corresponding correlations were plotted and manually checked for plausibility by removing artificial correlations.

All statistical computations were performed using R version 2.12.2 (http://www.r-project.org/), and Bioconductor version 2.7 [63]. An effect was considered statistically significant if the *P*-value was below 5%.

## SUPPLEMENTARY FIGUERS AND TABLES


