# Supplementary material for: miRNAs in multiple myeloma – a survival relevant complex regulator of gene expression
Source: Oncotarget. 2015 Oct 12;6(36):39165–83. doi: 10.18632/oncotarget.5381 (PMC4770764; doi:10.18632/oncotarget.5381)
Supplement: Supplementary file 1 [file oncotarget-06-39165-s001.pdf]

SUPPLEMENTARY FIGUERS AND TABLES

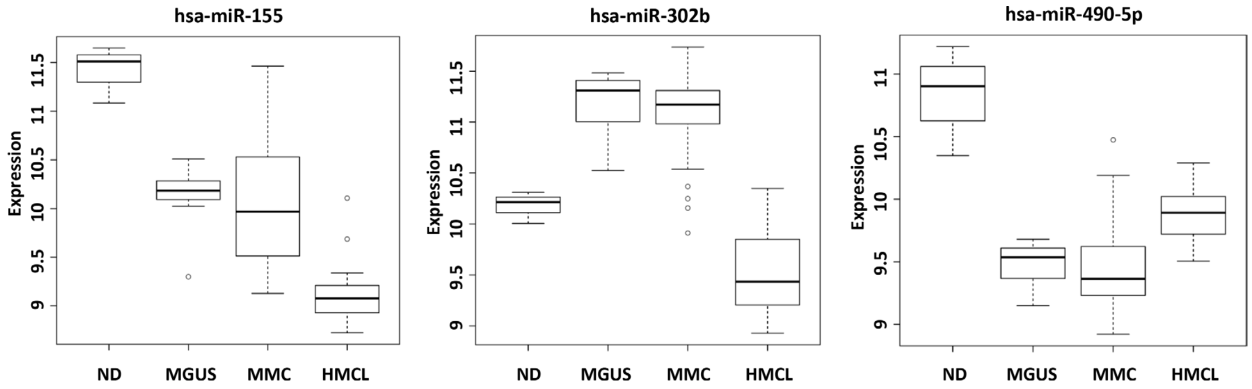

Supplementary Figure S1: Differential miRNA expression. Shown are three miRNAs, namely miR-155, miR-302b and miR-490-5p, which are differentially expressed between plasma cells from normal donors (ND), malignant plasma cells from MGUS and myeloma patients (MM), respectively, and human myeloma cell lines (HMCL).

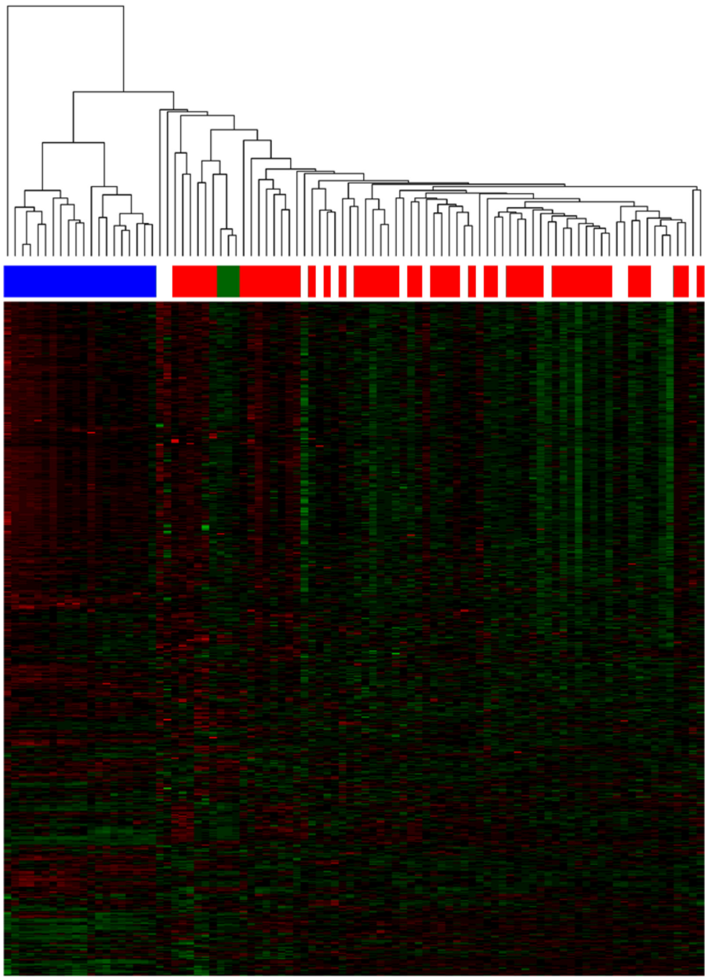

Supplementary Figure S2: Unsupervised clustering based on miRNA expression. The unsupervised clustering shows normal bone marrow plasma cells (depicted in green) clustering together in a sub-branch within the myeloma cell samples (depicted in white and red). Human myeloma cell lines (depicted in blue) clustering together in a separate branch. Patients with MGUS and early-stage myeloma (Salmon-Durie stage I; depicted in white) cluster within patients with late-stage myeloma (Salmon-Durie stage II and III; depicted in red), no sub-clusters can be found.

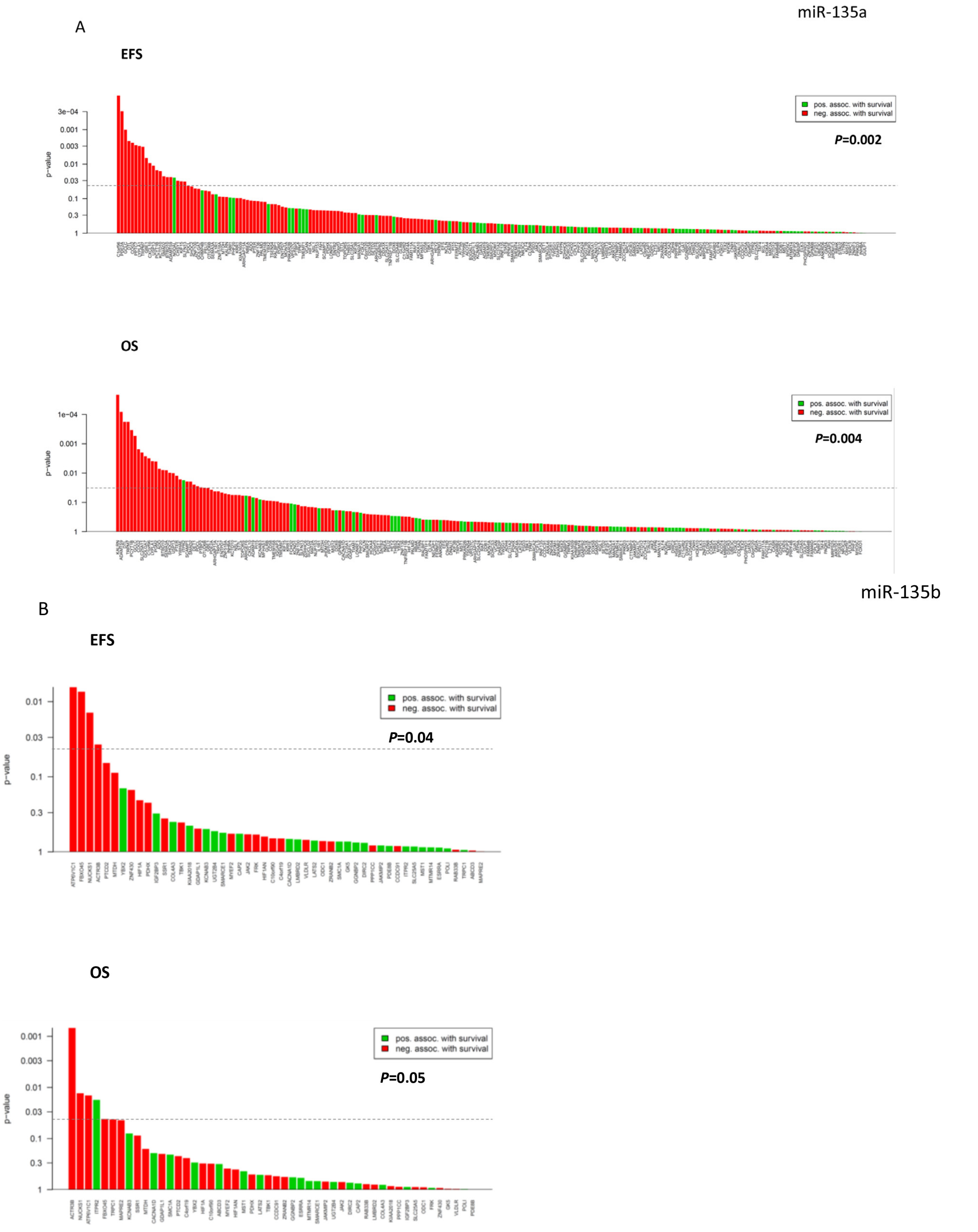

Supplementary Figure S3: Goeman global test. By using Goeman's global test, a significant association of the predicted target gene signatures of **A.** miR-135a, **B.**, miR-135b, (*Continued*)

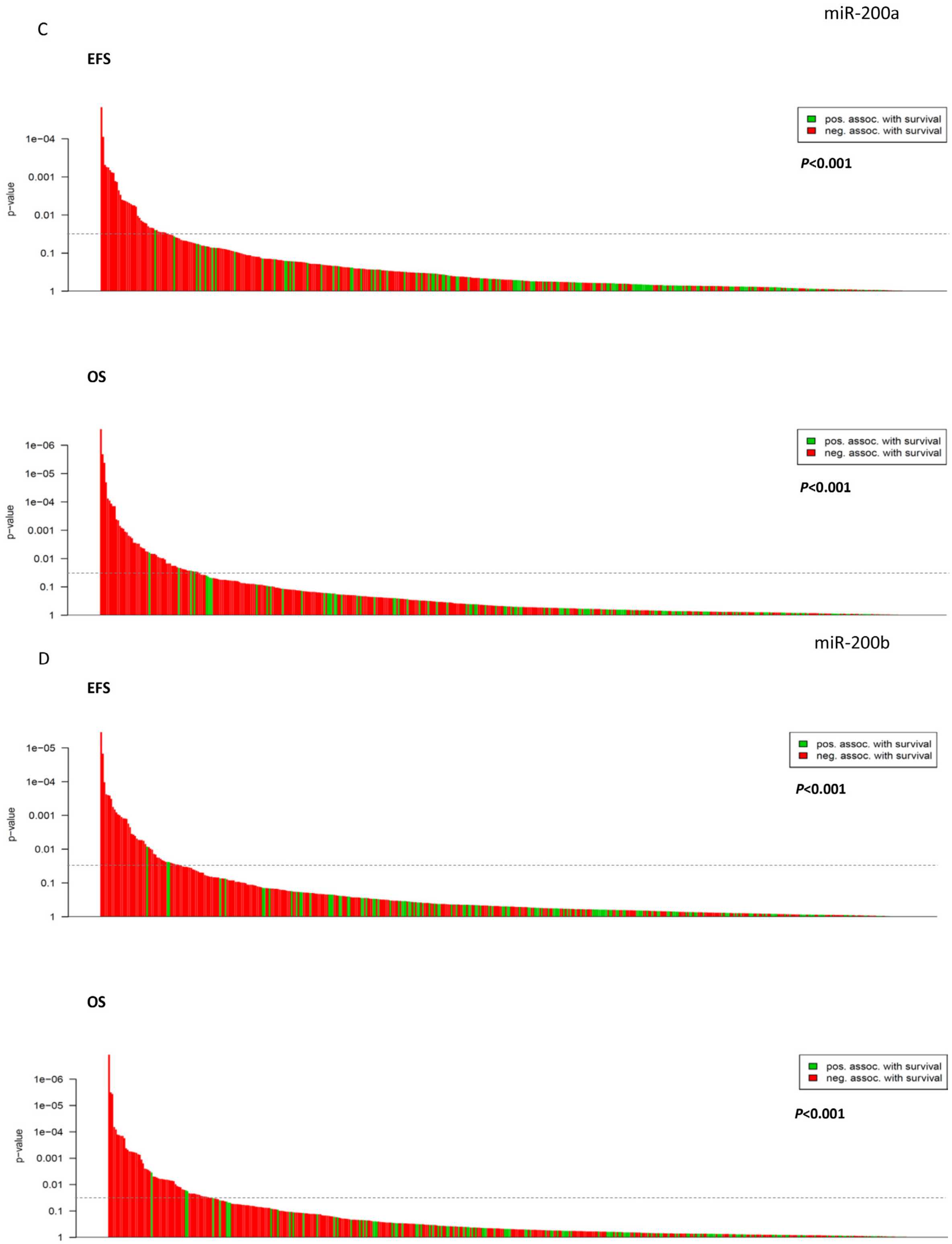

Supplementary Figure S3: (*Continued*) Goeman global test. C. miR-200a, **D.** miR-200a. (*Continued*)

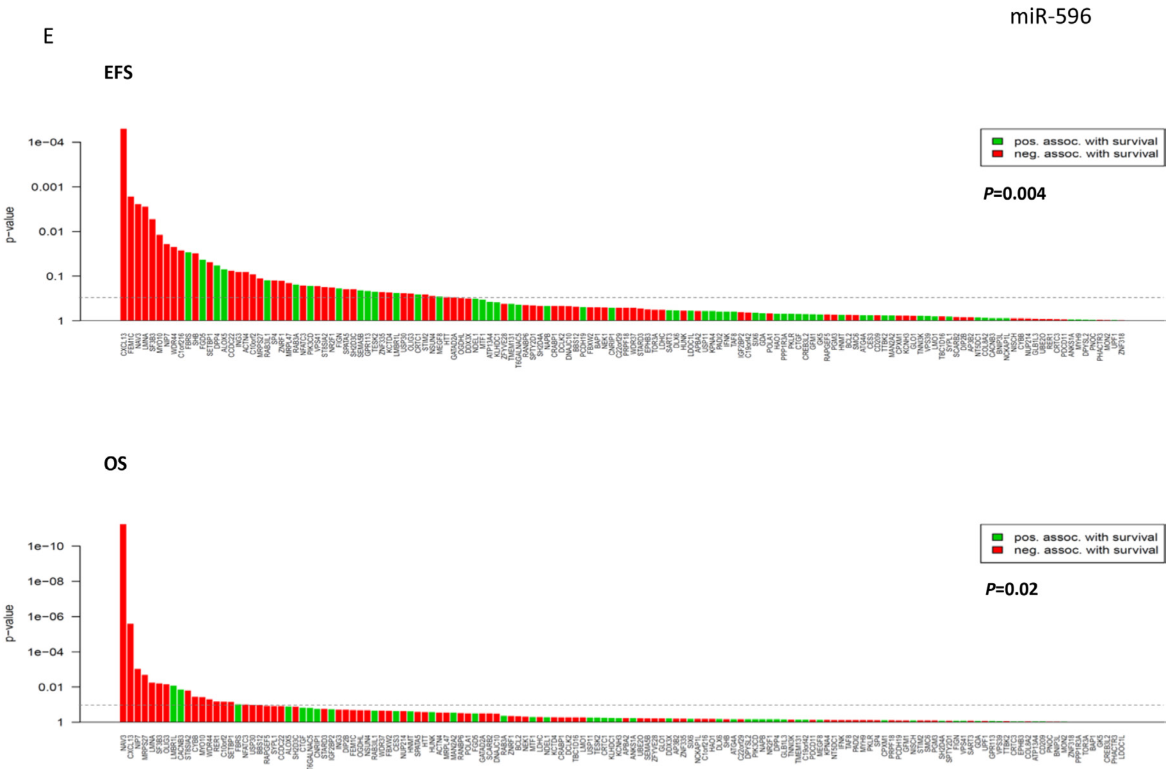

Supplementary Figure S3: (*Continued*) Goeman global test. E. miR-596 with survival could be found for both, event-free (EFS) and overall survival (OS). Target gene signatures for miR-135a, miR-135b, miR-200a, and miR-200b significantly predict for both, EFS (miR-135a, *P* = .002; miR-135b, *P* = .04; miR-200a, *P* < .001; miR-200b, *P* < .001) and OS (miR-135a, *P* = .004; miR-135b, *P* = .05; miR-200a, *P* < .001; miR-200b, *P* < .001). For each of these miRNAs, a high expression of the predicted target genes delineates a group with inferior survival (see Figure 3). *P*-value thresholds are shown as dashed lines.

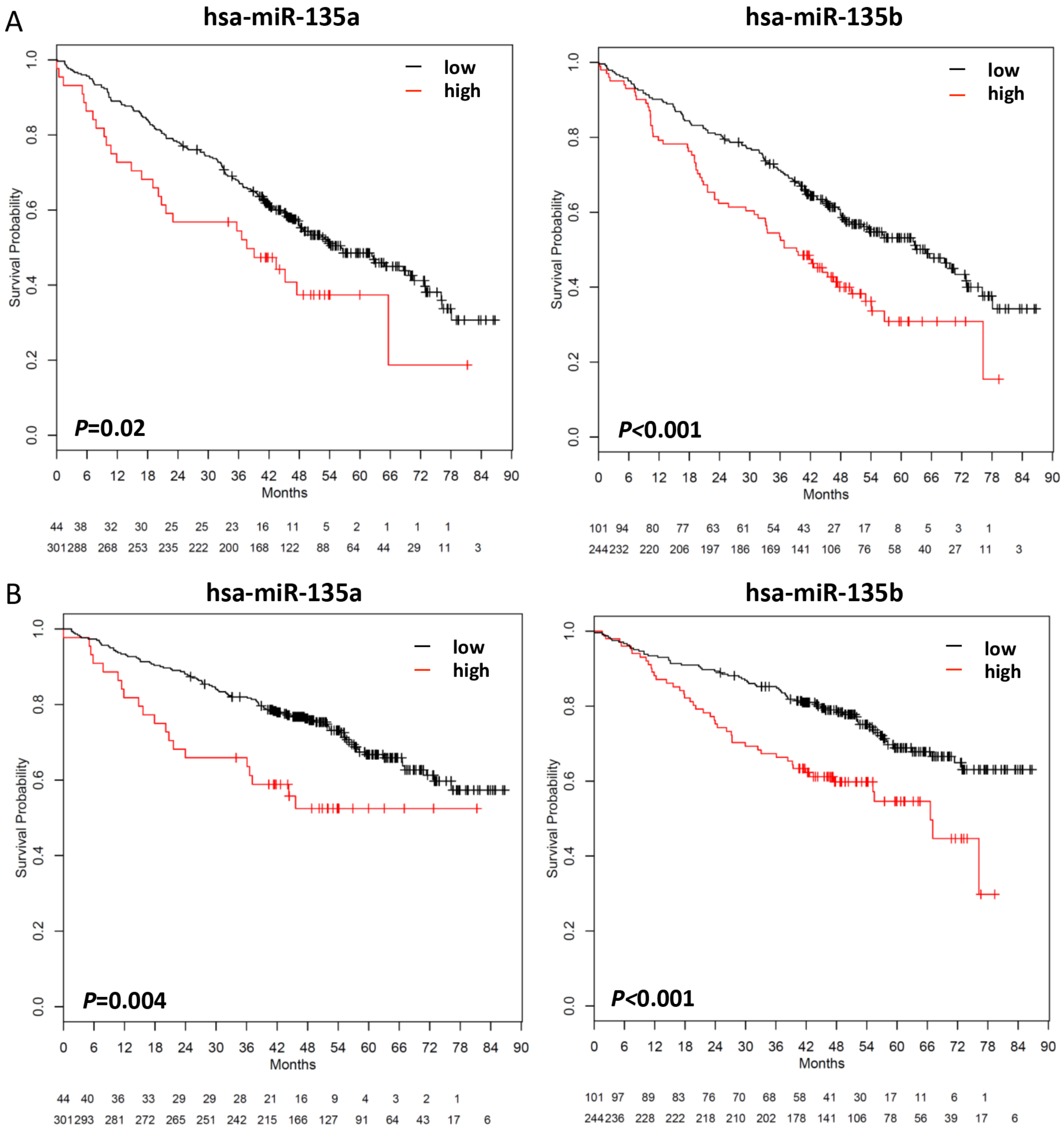
A
hsa-miR-135a
hsa-miR-135b
low
high
Survival Probability
Months
P=0.02
P<0.001
0 6 12 18 24 30 36 42 48 54 60 66 72 78 84 90
44 38 32 30 25 25 23 16 11 5 2 1 1 1
301 288 268 253 235 222 200 168 122 88 64 44 29 11 3
101 94 80 77 63 61 54 43 27 17 8 5 3 1
244 232 220 206 197 186 169 141 106 76 58 40 27 11 3
B
hsa-miR-135a
hsa-miR-135b
P=0.004
P<0.001
44 40 36 33 29 29 28 21 16 9 4 3 2 1
301 293 281 272 265 251 242 215 166 127 91 64 43 17 6
101 97 89 83 76 70 68 58 41 30 17 11 6 1
244 236 228 222 218 210 202 178 141 106 78 56 39 17 6

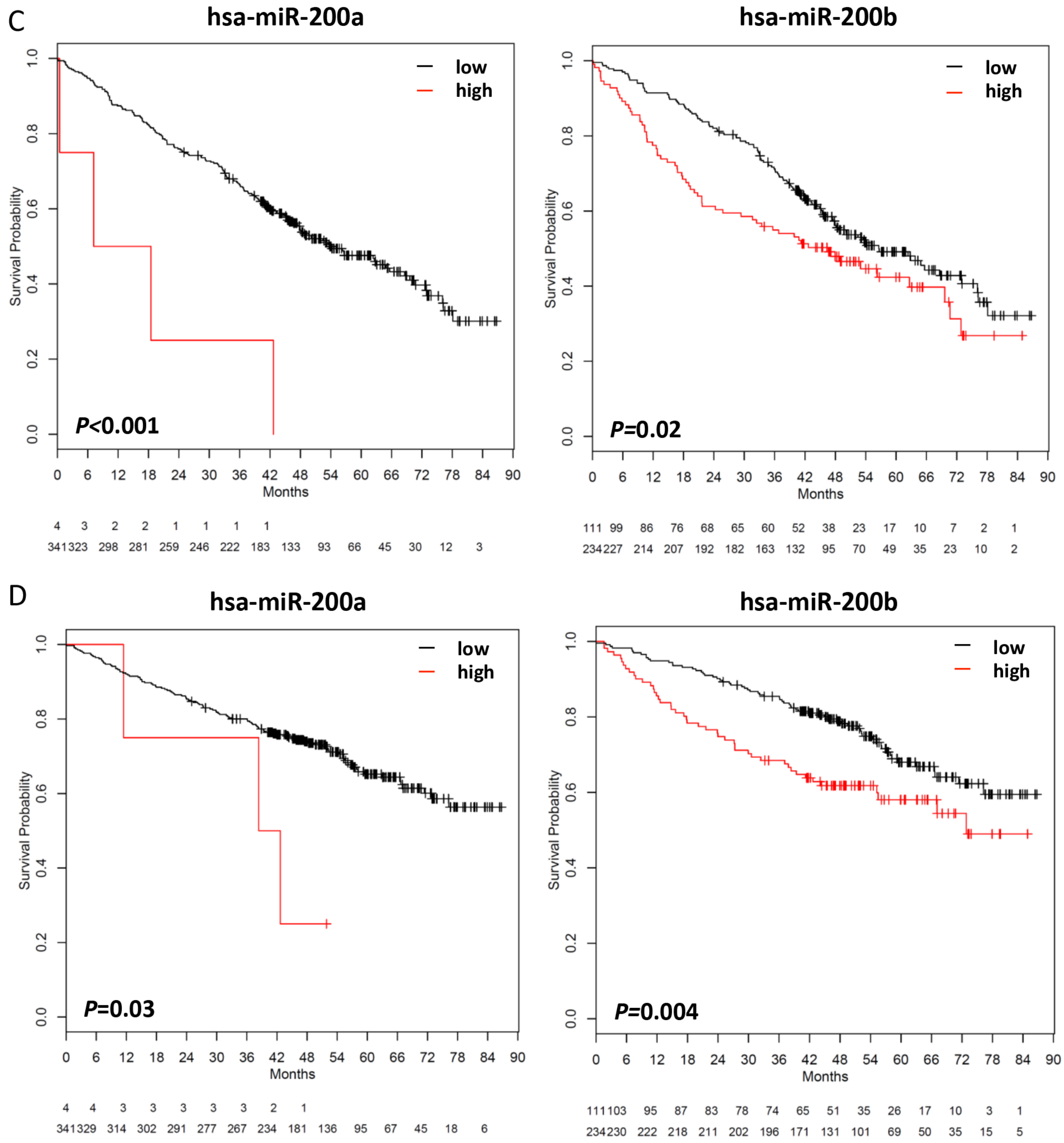

Supplementary Figure S4: Target gene signatures. By using miRWalk, predicted target genes for miR-135a, miR-135b, miR-200a, and miR-200b are significantly associated with **A, C.** event-free (EFS) and **B, D.** with overall survival (OS; Figure 4). For validation, data on an independent cohort of patients treated within the total therapy 2 protocol is shown. Data for miR-200a are based on only four patients in the high-risk group and have to be interpreted carefully.

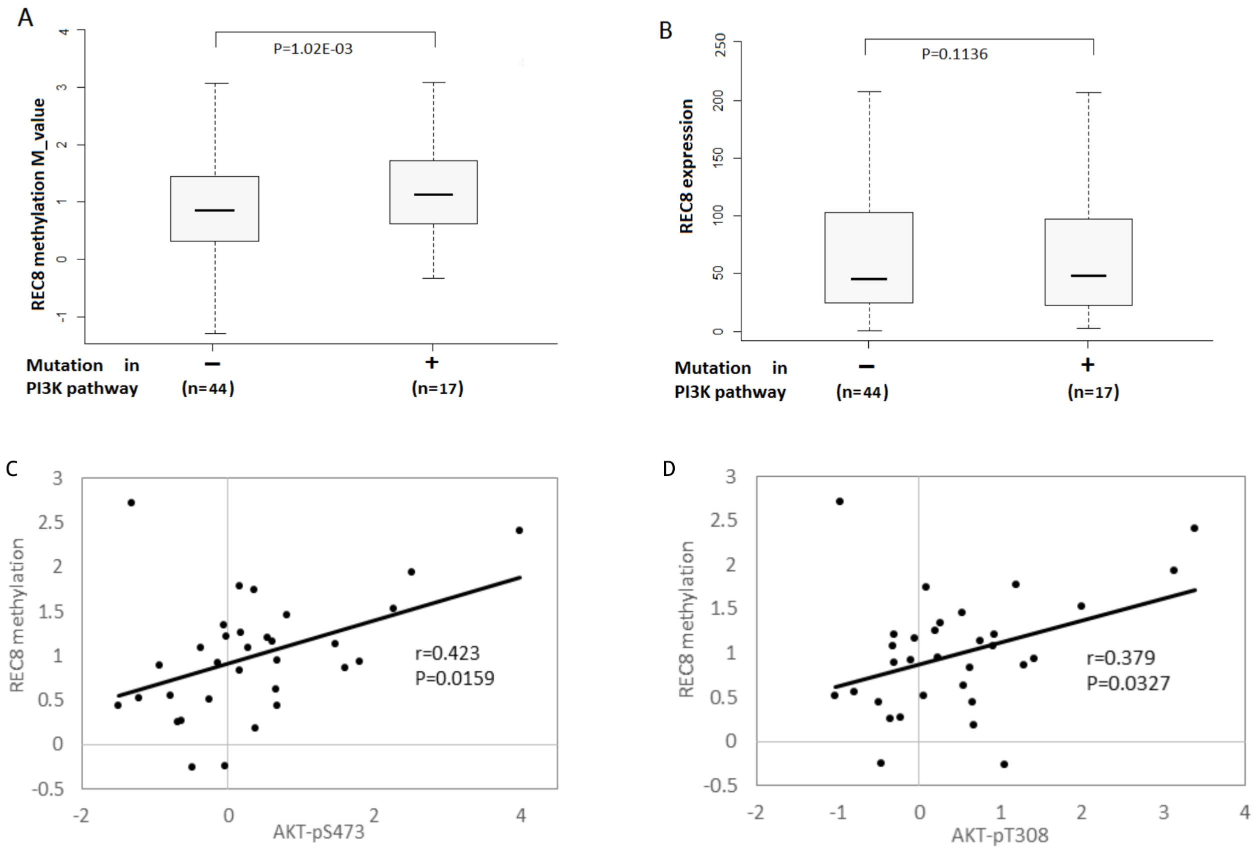

Supplementary Figure S5: Relationship between *REC8* methylation/expression and genetic alterations/signaling activities of the PI3K pathway in primary cutaneous melanoma in the TCGA database. A. Association between *REC8* hypermethylation and genetic alterations in the PI3K pathway in primary melanoma. The genetic alterations included mutations in *PIK3CA*, *PTEN* and the three *RAS* genes. **B.** Inverse association between the REC8 mRNA expression and the above genetic alterations in the PI3K pathway. **C.** Association between REC8 hypermethylation and AKT-pS473 phosphorylation in primary melanoma samples. **D.** Association between *REC8* hypermethylation and AKT- pT3083 phosphorylation in primary melanoma samples.

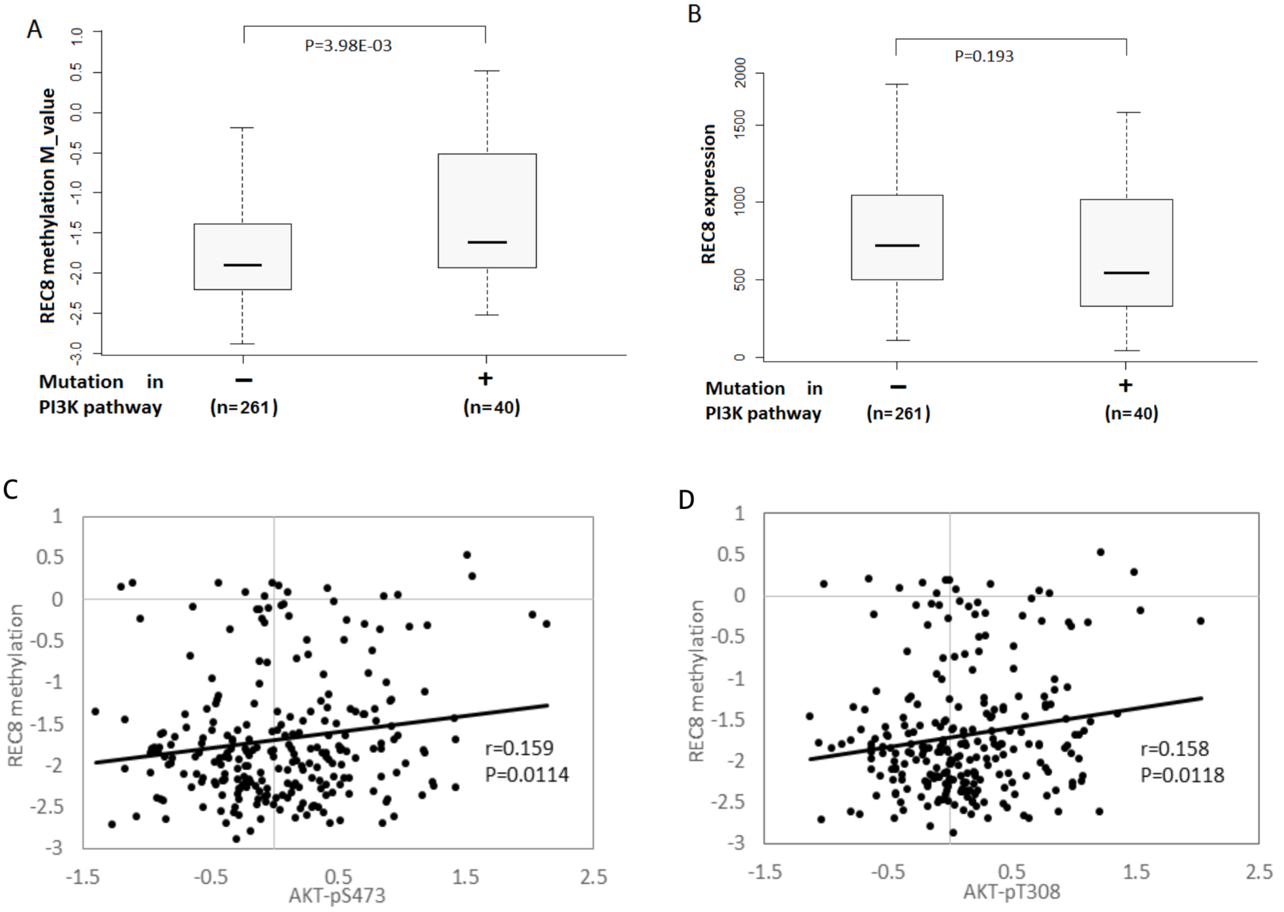

Supplementary Figure S6: Relationship between *REC8* methylation/expression and genetic alterations /signaling activities of the PI3K pathway in low-grade glioma in the TCGA database. A. Association between *REC8* hypermethylation and genetic alterations in PI3K pathway in glioma. The genetic alterations included mutations in *PIK3CA*, *PTEN* and the three *RAS* genes. **B.** Trend of inverse association between the *REC8* mRNA expression and the above genetic alterations in the PI3K pathway. **C.** Association between *REC8* hypermethylation and AKT-pS473 phosphorylation. **D.** Association between *REC8* hypermethylation and AKT-pT308 phosphorylation.

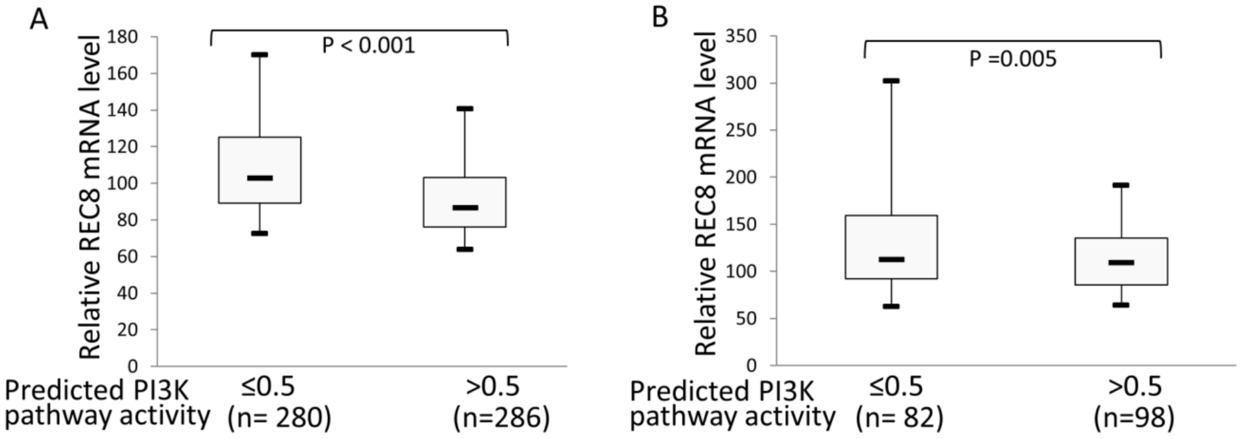

Supplementary Figure S7: Relationship between *REC8* expression and the PI3K pathway activity in colon cancer and melanoma in the database probeset: 218599. The expression data of *REC8* (probeset: 218599_at) in tumor samples were retrieved from the RMA-normalized microarray dataset. The predicted PI3K pathway activities (ranging from 0 to 1) of each tumor sample were calculated by BinReg algorithm as described in **Supplementary Materials and Methods.** The box-plot shows the five statistics (5th, 25th, 50th, 75th and 95th percentile) as described in the legend to **Fig. 5**). Randomization test was used to calculate the *p*-values for the difference of expression level in the two tumor groups. **A.** Box-Whisker plots of *REC8* expression level in colon cancer, which was significantly higher in the group with lower PI3K pathway activities (≤ 0.5) than in the group with higher PI3K pathway activities (> 0.5). **B**. Box-Whisker plots of *REC8* expression level in melanoma, which was significantly higher in the group with lower PI3K pathway activities (≤ 0.5) than in the group with higher PI3K pathway activities (> 0.5).

Supplementary Table S1: Overview over patient populations and samples used

Analysis		Details		miRNA cohort *n* =	GEP cohort *n* =
**miRNA profiling/ GEP**	**MMC**	HM-group		62	332
		LR-group		na	345
		**Sum**		**62**	**677**
	**other populations**	**MGUS**	HM-group	7	10
		**BMPC**	HM-group	3	22
		**HMCL**	HM-group	20	32
		**Sum**		**92**	**741**
**iFISH**	**MMC**	HM-group	t(4;14)	62 [6]	258 [18]
			t(11;14)	61 [13]	290 [12]
			t(14;16)	58 [3]	100 [6]
			1q21	61 [43]	242 [36]
			17p13	62 [6]	253 [13]
			13q14	62 [55]	262 [52]
**HDT**	**survival**	HM-group		53	247
		LR-group		na	345
		**Sum**		**53**	**592**

Shown is an overview over patient populations and samples used for miRNA profiling, gene expression profiling (GEP), and interphase fluorescence in situ hybridization (iFISH), as well as for survival analysis, i.e. patients treated with high-dose chemotherapy (HDT) and autologous stem cell transplantation. Numbers in squared brackets give the percentage of patients presenting with the respective chromosomal aberration. MMC, multiple myeloma cells; MGUS, monoclonal gammopathy of unknown significance; BMPC, bone marrow plasma cells; HMCL, human myeloma cell line (U266, RPMI-8226, LP-1, OPM-2, SK-MM-2, AMO-1, JJN-3, NCI-H929, KMS-12-BM, KMS-11, KMS-12-PE, KMS-18, KARPAS-620, MM1.S, JIM3, L363, MOLP-2, MOLP-8, EJM, and ANBL6 were used); HDT, high-dose chemotherapy; HM-group, Heidelberg/Montpellier-group; LR-group, Little Rock-group; na, not available.

Supplementary Table S2: Clinical data for patients undergoing high-dose chemotherapy and autologous stem cell transplantation

Characteristic	miRNA cohort	Gene expression profiling cohorts	
	n = 53	*n* = 247	*n* = 345
**Age**	62 [43–79]	67 [33-72]	57 [25–77]
**Monoclonal protein**			
IgA	10	144	193
IgG	34	56	93
Bence Jones	9	41	47
Asecretory	na	5	6
IgD	na	1	3
na	na	0	3
**Durie and Salmon stage**			
I	6	22	NA
II	5	37	NA
III	42	188	NA
**ISS stage**			
I	28	109	189
II	17	87	86
III	8	43	70
**Serum β2-microglobulin (mg/l)**	3.2 [0.9–40.5]	3.1 [1.3–53.6]	2.9 [1.0–38.7]
**Plasma cell infiltration (%)**	50 [9–97]	45 [2–100]	42 [4–98]

Clinical patient data for age, secreted monoclonal protein, myeloma stage according to Durie and Salmon and the International Staging System (ISS), serum-β2-microglobulin, and plasma cell infiltration. Median value and range are given. NA, not available.

Supplementary Table S3: Differential expression

miRNA	logFC	FC	Average expression	Adjusted *P*-value
**Samples from normal donors vs. MGUS-patients**				
miR-490-5p	1.35	2.5	9.59	< 0.001
miR-628-3p	1.33	2.5	10.28	0.02
miR-155	1.31	2.5	9.90	0.03
miR-516b	1.29	2.4	9.76	0.004
miR-500*	1.29	2.4	10.03	0.002
miR-553	1.20	2.3	9.48	0.01
miR-198	1.10	2.1	10.28	0.04
miR-659	0.99	2.0	11.44	0.04
miR-411	0.84	1.8	8.58	0.01
miR-615-3p	0.79	1.7	10.89	0.02
miR-450a	0.74	1.7	8.28	0.02
miR-645	0.67	1.6	9.10	0.01
miR-372	−0.59	−1.5	9.15	0.04
miR-346	−0.71	−1.6	10.21	0.04
miR-129*/129-3p	−0.89	−1.9	9.15	0.04
miR-634	−0.92	−1.9	10.21	0.01
miR-302b	−0.99	−2.0	10.74	0.01
let-7i	−1.06	−2.1	10.14	0.02
let-7f	−1.07	−2.1	10.02	0.04
miR-519d	−1.21	−2.3	11.70	0.04
**Samples from MM- vs. MGUS-patients**				
miR-200b*	−0.52	−1.4	9.86	0.02
miR-486-3p	−0.65	−1.6	11.24	0.007
miR-432	−0.78	−1.7	8.94	0.001

Shown are miRNAs that are differentially expressed between normal plasma cells and cells from MGUS-patients, as well as between malignant plasma cells from myeloma vs. MGUS-patients. miRNAs are listed according to the height of their fold change (FC).

Supplementary Table S4: Association between miRNAs and proliferation as well as high-risk scores

**Gene expression-based proliferation index**	
**miRNA**	**Adjusted *P*-value**
miR-93	0.006
miR-424	0.006
miR-492	0.006
miR-106b	0.01
miR-25	0.01
miR-623	0.02
miR-493*	0.02
miR-106a	0.02
miR-103	0.02
miR-17	0.03
**UAMS high-risk score**	
miR-596	0.003
miR-432*	0.01
miR-583	0.01
miR-135b	0.05
**IFM high-risk score**	
miR-93	< 0.001
miR-493*	< 0.001
miR-92b	< 0.001
miR-18a	0.003
miR-103	0.004
miR-20b	0.004
miR-25	0.005
miR-106a	0.005
miR-17	0.007
miR-106b	0.008
miR-515-5p	0.01
miR-125b-1*	0.01
miR-365	0.01
miR-107	0.01
miR-19a	0.02
miR-297	0.02
miR-205	0.02
miR-20a	0.02
miR-615-3p	0.02

(*Continued*)

IFM high-risk score miRNA	Adjusted P-value
miR-520b/520c-3p	0.03
miR-301b	0.03
miR-216b	0.03
miR-142-5p	0.03
miR-424	0.03
miR-583	0.04
miR-584	0.04
miR-623	0.04
miR-659	0.04
miR-32	0.04
miR-519e*	0.04
miR-425	0.05
miR-934	0.05

Shown are miRNAs that are significantly associated with the gene expression-based proliferation index, the UAMS-, and the IFM high-risk score, respectively.

Supplementary Table S5: miRNAs correlated with proliferation and high-risk scores

Gene expression-based proliferation index		
**miRNA**	**Correlation (r)**	**Adjusted *P*-value**
miR-93	0.53	0.006
miR-424	0.53	0.006
miR-106b	0.50	0.01
miR-25	0.50	0.01
miR-106a	0.46	0.02
miR-103	0.46	0.02
miR-17	0.45	0.03
miR-493*	−0.47	0.02
miR-623	−0.48	0.02
miR-492	−0.52	0.006
**UAMS high-risk score**		
miR-596	0.32	0.003
miR-432*	0.27	0.01
miR-583	0.26	0.01
miR-135b	0.21	0.05

(*Continued*)

IFM high-risk score		
**miRNA**	**Correlation (r)**	**Adjusted *P*-value**
miR-103	0.62	< 0.001
miR-107	0.59	< 0.001
miR-93	0.59	< 0.001
miR-106a	0.56	0.001
miR-17	0.55	0.001
miR-365	0.55	0.001
miR-18a	0.54	0.001
miR-424	0.54	0.001
miR-25	0.53	0.001
miR-106b	0.53	0.002
miR-142-5p	0.52	0.002
miR-20b	0.51	0.002
miR-21	0.48	0.006
miR-484	0.48	0.007
miR-20a	0.48	0.007
miR-520b/520c-3p	0.46	0.01
miR-324-5p	0.46	0.01
miR-19a	0.46	0.01
miR-142-3p	0.45	0.01
miR-24	0.44	0.02
miR-301a	0.43	0.02
miR-193a-3p	0.41	0.03
miR-191	0.41	0.03
miR-515-5p	0.41	0.03
miR-574-3p	0.41	0.03
miR-143	0.41	0.03
miR-32	0.40	0.04
miR-590-5p	0.39	0.04
miR-504	0.38	0.05
miR-299-3p	−0.39	0.04
miR-519e*	−0.40	0.04
miR-492	−0.40	0.04
miR-615-3p	−0.40	0.04
miR-503	−0.41	0.03
miR-125b-1*	−0.41	0.03
miR-208	−0.42	0.03

(*Continued*)

IFM high-risk score		
**miRNA**	**Correlation (r)**	**Adjusted *P*-value**
miR-584	−0.44	0.02
miR-493*	−0.51	0.003

Shown are miRNAs that are significantly correlated with the gene expression-based proliferation index, the UAMS-, and the IFM high-risk score, respectively.

Supplementary Table S6: Differential expression

A

Gene expression-based proliferation index: GPI low vs. GPI high				
**miRNA**	**logFC**	**FC**	**Average expression**	**Adjusted *P*-value**
miR-103	−0.64	−1.6	9.24	0.01
miR-93	−0.70	−1.6	8.36	0.01
miR-18a	−0.70	−1.6	8.68	0.02
miR-106b	−0.73	−1.7	9.52	0.01
miR-424	−1.03	−2.0	8.32	0.01
miR-20b	−1.05	−2.1	9.67	0.02
miR-17	−1.10	−2.1	10.26	0.01
miR-106a	−1.20	−2.3	10.03	0.01
**UAMS high-risk: low-risk vs. high-risk**				
miR-135b	−0.52	−1.4	8.29	0.03
miR-596	−0.67	−1.6	9.52	0.002
miR-432*	−0.79	−1.7	10.15	0.006
miR-583	−0.84	−1.8	12.73	0.007
**IFM high-risk score: low-risk vs. high-risk**				
miR-493*	0.51	1.4	9.96	< 0.001
miR-584	0.44	1.4	14.07	0.04
miR-518e*/519a*/519b-5p/519c-5p/522*/523*	0.43	1.3	10.14	0.04
miR-659	0.41	1.3	11.26	0.04
miR-623	0.39	1.3	12.31	0.04
miR-125b-1*	0.35	1.3	10.24	0.01
miR-519e*	0.35	1.3	12.08	0.04
miR-635	0.33	1.3	9.87	0.05
miR-615-3p	0.29	1.2	10.70	0.02
miR-934	0.28	1.2	10.45	0.05
miR-205	−0.20	−1.1	8.37	0.03
miR-520b/520c-3p	−0.21	−1.2	8.19	0.04
miR-216b	−0.23	−1.2	8.14	0.04

(*Continued*)

A

IFM high-risk score: low-risk vs. high-risk

miRNA	logFC		Average expression	Adjusted P-value
miR-297	−0.25	−1.2	8.10	0.02
miR-301b	−0.28	−1.2	8.20	0.03
miR-425	−0.30	−1.2	8.62	0.04
miR-515-5p	−0.32	−1.2	8.41	0.01
miR-103	−0.42	−1.3	9.24	0.003
miR-32	−0.42	−1.3	8.61	0.04
miR-106b	−0.44	−1.4	9.52	0.006
miR-92b	−0.45	−1.4	8.43	< 0.001
miR-93	−0.48	−1.4	8.36	< 0.001
miR-18a	−0.49	−1.4	8.68	0.002
miR-25	−0.51	−1.4	8.99	0.004
miR-424	−0.54	−1.5	8.32	0.03
miR-107	−0.57	−1.5	9.40	0.01
miR-20a	−0.62	−1.5	9.94	0.02
miR-17	−0.70	−1.6	10.26	0.004
miR-142-5p	−0.71	−1.6	8.73	0.02
miR-583	−0.73	−1.7	12.73	0.03
miR-20b	−0.73	−1.7	9.67	0.003
miR-365	−0.75	−1.7	9.47	0.01
miR-19a	−0.76	−1.7	11.22	0.01
miR-106a	−0.80	−1.7	10.03	0.003

B

Translocation t(4;14): none vs. t(4;14)

miRNA	logFC	FC	Average expression	Adjusted *P*-value
miR-596	−0.69	−1.6	9.52	0.004
miR-135a	−0.76	−1.7	8.39	0.01
miR-432*	−0.91	−1.9	10.12	0.004
**Translocation t(11;14): none vs. t(11;14)**				
miR-138	1.04	2.1	9.49	0.01
miR-519e	0.68	1.6	13.02	0.03
miR-584	0.58	1.5	14.05	0.05
miR-200c*	0.56	1.5	11.01	0.02
miR-519d	0.53	1.4	11.97	0.04
miR-361-5p	0.50	1.4	11.29	0.01

(*Continued*)

B				
**Translocation t(11;14): none vs. t(11;14)**				
**miRNA**	**logFC**	**FC**	**Average expression**	**Adjusted P-value**
miR-324-5p	0.45	1.4	10.26	0.05
miR-615-3p	0.41	1.3	10.69	0.02
miR-16-1*	−0.35	−1.3	8.42	0.03
miR-517c	−0.37	−1.3	8.61	0.04
miR-30e*	−0.44	−1.4	8.79	0.05
miR-15b*	−0.45	−1.4	8.33	0.03
miR-15a*	−0.47	−1.4	8.51	0.01
miR-27a*	−0.51	−1.4	8.66	0.02
miR-32	−0.52	−1.4	8.62	0.05
miR-147	−0.53	−1.4	8.42	0.02
miR-140-5p	−0.54	−1.5	8.63	0.02
miR-193a-3p	−0.60	−1.5	8.84	0.04
miR-374a	−0.66	−1.6	9.44	0.04
miR-193b	−0.66	−1.6	9.38	0.02
miR-223	−0.72	−1.6	8.78	0.01
miR-15b	−0.72	−1.6	9.85	0.02
miR-15a	−0.92	−1.9	9.69	0.01
miR-22*	−0.94	−1.9	9.17	0.01
**Deletion 13q: none vs. del13q**				
miR-767-5p	0.45	1.4	9.02	0.03
miR-23a	−0.47	−1.4	11.21	0.002
miR-23b	−0.49	−1.4	11.39	0.002
**Hyperdiploidie: none vs. hyperdiploid**				
miR-21	1.07	2.1	10.71	0.02
miR-22	0.83	1.8	10.04	0.003
miR-125b	0.72	1.6	8.72	0.03
−miR-374b	0.45	1.4	9.18	0.02
hsa-miR-93	0.34	1.3	8.42	0.02

Shown are (A) miRNAs that are differentially expressed between low-risk and high-risk according to the gene expression-based proliferation index, as well as the UAMS- and the IFM-score. (B) Shows those miRNAs that are differentially expressed between samples with vs. without the respective chromosomal aberration. miRNAs are listed according to the height of their fold change (FC).

Supplementary Table S7: Prognostic impact of mRNAs significantly correlated with miRNA

Gene symbol	mRNA probeset	miRNA	EFS			OS		
			HR	[95 % CI]	adj. *P*-value	HR	[95 % CI]	adj.*P*-value
KCTD11	235857_at	let-7f	0.966	[0.788;1.185]	0.7	0.983	[0.741;1.304]	0.9
EXTL2	209537_at	let-7g	0.989	[0.892;1.096]	0.8	1.03	[0.892;1.191]	0.7
XRCC4	205071_x_at	let-7i	0.93	[0.846;1.022]	0.1	0.958	[0.844;1.087]	0.5
ITPR1	203710_at	miR-19b	1.044	[0.968;1.126]	0.3	1.048	[0.946;1.16]	0.4
SELM	226051_at	miR-19b	0.941	[0.856;1.035]	0.2	0.859	[0.769;0.96]	0.007
GRN	200678_x_at	miR-100	0.927	[0.787;1.092]	0.4	0.905	[0.718;1.14]	0.4
SIN3A	225135_at	miR-103	1.149	[1.036;1.276]	0.009	1.236	[1.079;1.417]	0.002
GRN	200678_x_at	miR-103	0.927	[0.787;1.092]	0.4	0.905	[0.718;1.14]	0.4
SELM	226051_at	miR-103	0.941	[0.856;1.035]	0.2	0.859	[0.769;0.96]	0.007
FBXO11	219208_at	miR-106b	1.144	[1.017;1.287]	0.03	1.176	[1.012;1.367]	0.03
TMPO	209754_s_at	miR-106b	1.083	[0.999;1.173]	0.05	1.091	[0.981;1.214]	0.1
BUB1	209642_at	miR-106b	1.164	[1.094;1.238]	<0.001	1.198	[1.107;1.296]	<0.001
MCM4	222036_s_at	miR-106b	1.256	[1.105;1.429]	0.001	1.32	[1.107;1.575]	0.002
SIN3A	225135_at	miR-106b	1.149	[1.036;1.276]	0.009	1.236	[1.079;1.417]	0.002
RPS6KA3	203843_at	miR-106b	1.222	[1.059;1.41]	0.006	1.495	[1.238;1.806]	<0.001
BUB1B	203755_at	miR-106b	1.146	[1.066;1.233]	<0.001	1.195	[1.084;1.318]	<0.001
GRN	200678_x_at	miR-106b	0.927	[0.787;1.092]	0.4	0.905	[0.718;1.14]	0.4
HIST1H2AC	215071_s_at	miR-186	1.091	[0.976;1.219]	0.1	1.17	[1.004;1.363]	0.04
HIST1H2AC	215071_s_at	miR-374a	1.091	[0.976;1.219]	0.1	1.17	[1.004;1.363]	0.04
TNIP2	232160_s_at	miR-424	1.245	[1.053;1.473]	0.01	1.418	[1.157;1.738]	0.001
GINS2	221521_s_at	miR-424	1.261	[1.119;1.42]	<0.001	1.389	[1.19;1.622]	<0.001
EPB41L2	201719_s_at	miR-602	0.938	[0.82;1.074]	0.4	0.879	[0.736;1.049]	0.2
WEE1	212533_at	miR-623	1.136	[1.044;1.236]	0.003	1.188	[1.065;1.326]	0.002
GCLM	236140_at	miR-623	1.12	[1.011;1.239]	0.03	1.062	[0.929;1.215]	0.4
MCM6	201930_at	miR-623	1.201	[1.057;1.364]	0.005	1.362	[1.149;1.614]	<0.001
STRN	236388_at	miR-623	1.396	[1.137;1.715]	0.001	1.502	[1.171;1.926]	0.001
RAPH1	225188_at	miR-623	1.089	[1.027;1.154]	0.004	1.055	[0.974;1.143]	0.2
ACTR2	200729_s_at	miR-623	1.153	[0.973;1.365]	0.1	1.235	[0.976;1.564]	0.08
RMI2	226456_at	miR-623	1.165	[1.063;1.278]	0.001	1.21	[1.077;1.359]	0.001

Shown are mRNAs that we found to be correlated with miRNAs with a correlation coefficient $r \geq 0.6$ or $r \leq -0.6$ (see Table 3) and their impact on event-free (EFS) and overall survival (OS) for patients treated within the total therapy 2 protocol for validation purposes. HR, hazard ratio; CI, confidence interval.
